# Molecular determinants and mechanism for antibody cocktail preventing SARS-CoV-2 escape

**DOI:** 10.1038/s41467-020-20789-7

**Published:** 2021-01-20

**Authors:** Zhiqiang Ku, Xuping Xie, Edgar Davidson, Xiaohua Ye, Hang Su, Vineet D. Menachery, Yize Li, Zihao Yuan, Xianwen Zhang, Antonio E. Muruato, Ariadna Grinyo i Escuer, Breanna Tyrell, Kyle Doolan, Benjamin J. Doranz, Daniel Wrapp, Paul F. Bates, Jason S. McLellan, Susan R. Weiss, Ningyan Zhang, Pei-Yong Shi, Zhiqiang An

**Affiliations:** 1 Texas Therapeutics Institute, Brown Foundation Institute of Molecular Medicine, University of Texas Health Science Center at Houston, Houston, TX 77030 USA; 2 Department of Biochemistry and Molecular Biology, Institute for Human Infection and Immunity, Sealy Institute for Vaccine Sciences, Sealy Center for Structural Biology & Molecular Biophysics, Department of Pharmacology & Toxicology, University of Texas Medical Branch, Galveston, TX 77555 USA; 3 Integral Molecular, Philadelphia, Pennsylvania, PA 19104 USA; 4 Department of Microbiology & Immunology, University of Texas Medical Branch, Galveston, TX 77555 USA; 5 Department of Microbiology, Perelman School of Medicine, University of Pennsylvania, Philadelphia, PA 19104 USA; 6 Department of Molecular Biosciences, University of Texas at Austin, Austin, TX 78712 USA

**Keywords:** Antibodies, SARS-CoV-2

## Abstract

Antibody cocktails represent a promising approach to prevent SARS-CoV-2 escape. The determinants for selecting antibody combinations and the mechanism that antibody cocktails prevent viral escape remain unclear. We compared the critical residues in the receptor-binding domain (RBD) used by multiple neutralizing antibodies and cocktails and identified a combination of two antibodies CoV2-06 and CoV2-14 for preventing viral escape. The two antibodies simultaneously bind to non-overlapping epitopes and independently compete for receptor binding. SARS-CoV-2 rapidly escapes from individual antibodies by generating resistant mutations in vitro, but it doesn’t escape from the cocktail due to stronger mutational constraints on RBD-ACE2 interaction and RBD protein folding requirements. We also identified a conserved neutralizing epitope shared between SARS-CoV-2 and SARS-CoV for antibody CoV2-12. Treatments with CoV2-06 and CoV2-14 individually and in combination confer protection in mice. These findings provide insights for rational selection and mechanistic understanding of antibody cocktails as candidates for treating COVID-19.

## Abstract (web summary)

Antibody cocktails represent a promising approach to prevent SARS-CoV-2 escape. Here, Ku et al., identify SARS-CoV-2 neutralizing antibodies from a phage library and identify an antibody combination that prevents viral escape and protects mice from viral challenge.

## Introduction

Coronavirus disease 2019 (COVID-19) is caused by a novel severe acute respiratory syndrome coronavirus 2 (SARS-CoV-2). Efforts to develop vaccines and therapies in response to the global pandemic of COVID-19 proceed at an unprecedented scale and pace^1,2^. SARS-CoV-2 uses its spike protein (S) to hijack the cellular receptor angiotensin-converting enzyme 2 (ACE2) and initiate infection^3^. The S protein is a homotrimer; each monomer comprises an N-terminal S1 subunit responsible for receptor binding and a C-terminal S2 subunit responsible for membrane fusion^3–5^. The receptor-binding domain (RBD) of S directly interacts with ACE2, and is a primary target for neutralizing antibodies^3^. In COVID-19 patients and vaccinated animals, RBD-directed antibodies are dominant in the total neutralizing antibody response^6,7^. These findings highlights the importance of targeting RBD for antibody intervention.

The RBD comprises a core structure, which serves as a scaffold, and a receptor-binding motif (RBM), which mediates direct contact with ACE2 (ref. ^8^). Because of its critical function, the RBM contains antigenic sites for eliciting potent neutralizing antibodies^8^. Recently, several highly potent neutralizing monoclonal antibodies (mAbs) targeting the RBM have been isolated from COVID-19 patients^9–17^. In addition, a number of core region-directed antibodies, which are cross-reactive to SARS-CoV, have also been identified^18–20^. Despite its critical function, the RBD of SARS-CoV-2 continues to evolve, as evidenced by the emergence of viral isolates with diverse amino acid changes in both the RBM and core regions of the RBD^21^. A substantial number of mutations in the RBD are well tolerated with the binding to ACE2 maintained^22,23^. This raises the concern of neutralization escape, which may severely limit the therapeutic potential of neutralizing mAbs. Using a recombinant Vesicular Stomatitis Virus (VSV) expressing the S of SARS-CoV-2, a recent study demonstrated the rapid emergence of escape mutants in the presence of RBD-specific single mAbs, regardless of their neutralizing activities^24^. Several RBD-directed mAbs have entered clinic trials (NCT04425629, NCT04452318, NCT04427501, and NCT04441918). Their ability to prevent neutralization escape of SARS-CoV-2, particularly the authentic live virus, has yet to be determined.

Antibody cocktail approaches have shown promise in avoiding neutralization escape by viruses in vitro^25,26^. An antibody cocktail for treating the Ebola virus disease has demonstrated clinic success^27^. Recently, a dual-antibody cocktail (REG10987+REG10933) for SARS-CoV-2 has entered phase 2/3 clinical trials. This antibody cocktail is capable of preventing mutational escape as evaluated in cell culture using the VSV–SARS-CoV-2 S recombinant virus^24^. Another antibody cocktail COV2-2130+COV2-2196, which exhibited neutralization synergy and animal protection^15^, has entered phase 1 clinical trial (NCT04507256). Other antibody cocktails, including BD-368-2+BD-629 (ref. ^28^) and B38+H4 (ref. ^29^) have also been evaluated for neutralization activities. However, the molecular determinants optimal for cocktail mAbs and the mechanism of preventing viral escape remain poorly understood. These key challenges impede the development of mAb cocktails for SARS-CoV-2. Only certain mAb combinations can effectively prevent viral escape^24^. This result suggests the importance of individual mAbs in a combination targeting different vulnerable sites. Therefore, identifying effective mAb cocktails, defining the molecular determinants on the RBD, and elucidating the mechanism of preventing viral escape are critically important to accelerate the development of effective cocktail mAb therapies for COVID-19.

Here, we show a cocktail of two mAbs (CoV2-06+CoV2-14) that target the RBD and cooperate with each other to prevent escape mutations. The two mAbs bind to non-overlapping epitopes of the RBD and independently block RBD and ACE2 interaction. The cocktail prevents SARS-CoV-2 escape mutations through a mechanism of imposing stronger mutational constraints on the RBD than individual mAbs. Individual mAbs and the cocktail confer protections against SARS-CoV-2 infection in mice. Overall, our comprehensive study provides important molecular insights for the development of antibody cocktail therapies for COVID-19.

## Results

### Isolation of RBD-directed human mAbs with potent neutralization of SARS-CoV-2

**Fig. 1:**
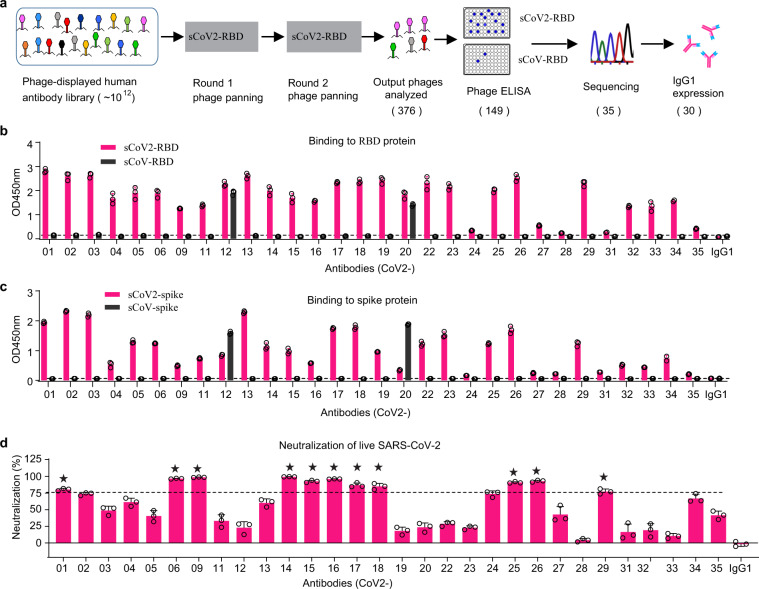
Isolation of RBD-directed human mAbs with neutralizing activates against SARS-CoV-2. **a** Flowcharts of the scFv phage library panning and mAb selection process. **b**, **c** ELISA binding of purified mAbs to the RBD proteins (**b**) and the S proteins (**c**) of SARS-CoV-2 and SARS-CoV. The dashed line is 2× the OD450nm of a control IgG1 and as a cut-off for binders. **d** Neutralization of live SARS-CoV-2 by the antibodies at 10 µg/ml. The dashed line indicates a 75% neutralization. The stars indicate the 11 mAbs with neutralization above 75%. Error bars indicate SD of triplicates.

Since the RBD of SARS-CoV-2 S protein is a crucial antibody target, we focused on isolating RBD-specific mAbs from a single-chain variable fragment (scFv) phage display antibody library. We generated a highly purified Fc-tagged RBD of SARS-CoV-2 (sCoV2-RBD) protein as bait for phage panning. We also prepared the RBD of SARS-CoV (sCoV-RBD) for the evaluation of cross-reactivity (Supplementary Fig. 1a, b). The purified RBD proteins bind ACE2 with high affinity (Supplementary Fig. 1c), indicating that these proteins retain the correct conformations. We used sequential panning rounds of a highly diverse naïve scFv phage library with increased stringency to select sCoV2-RBD-bound phages. The output phages were analyzed for antigen binding by ELISA. Unique scFv clones were identified by sequencing and converted to full human immunoglobulin G1 (IgG1). After the panning and selection process, 30 mAbs were obtained (Fig .1a). Among the 30 sCoV2-RBD binding mAbs, two mAbs (CoV2-12 and CoV2-20) show cross-binding to sCoV-RBD (Fig. 1b). All 30 mAbs also bind to the trimeric prefusion S protein of SARS-CoV-2 (Fig. 1c). Cross-binding of CoV2-12 and CoV2-20 to the S of SARS-CoV was confirmed (Fig.1c). We next screened the 30 mAbs for neutralization of a live SARS-CoV-2 virus engineered with the mNeonGreen marker^30^. Among them, 11 mAbs achieved >75% neutralization at 10 µg/ml (NT_75_ <10 µg/ml) and the remaining 19 mAbs exhibited <75% neutralization at 10 µg/ml (NT_75_ >10 µg/ml) (Fig. 1d).

We analyzed the germline genes for the variable heavy (VH) and variable light (VL) regions of the 30 mAbs (Supplementary Fig. 2). The VHs fall within four different gene classes: *VH1*, *VH3*, *VH4*, and *VH6*. The VHs originated from 13 gene alleles. Notably, the VH of CoV2-14 and CoV2-15 had 100% homology to the original human germline sequence, indicating no somatic mutations (Supplementary Fig. 2a). The VLs also fall within four gene classes: the *VK1*, *VL1*, *VL2*, and *VL3*. The VLs originated from 12 gene alleles (Supplementary Fig. 2a). The VLs show a bias toward the lambda over kappa usage (Supplementary Fig. 2b); the typical distribution of human IgG antibodies has a 2:1 ratio of kappa : lambda light chain usage. We further divided the 30 mAbs into two groups based on their NT_75_ values (Fig. 1d). We compared the gene usage and CDR3 length of these two groups (Supplementary Fig. 2c, d). Significantly, the group of mAbs with NT_75_ <10 μg/ml had a bias toward lambda light chain usage (91% vs. 42% for mAbs with NT_75_ >10 μg/ml) (Supplementary Fig. 2c). No significant difference between the CDR3 lengths of the two groups was detected (Supplementary Fig. 2d).

### Identification of neutralizing mAbs with simultaneous binding to RBD

**Fig. 2:**
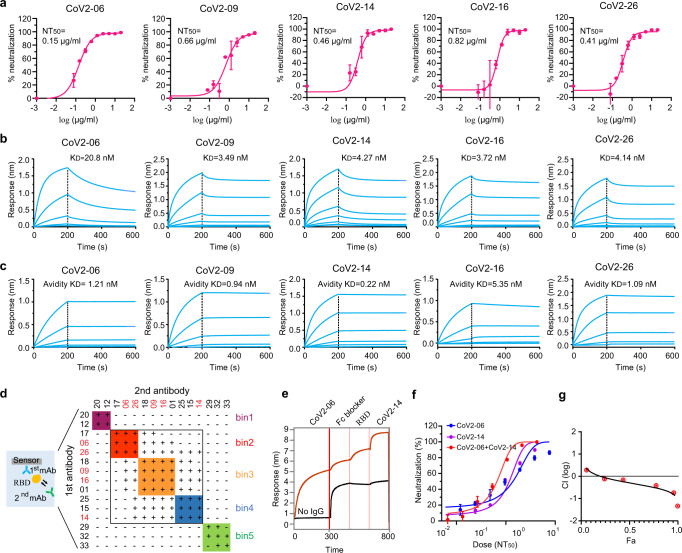
Identification of CoV2-06 and CoV2-14 as two neutralizing mAbs suitable for cocktail. **a** Neutralization titration curves of the top five mAbs with 50% neutralization titer (NT_50_) below 1 µg/ml. Each data point is the mean ± SD of two replicates. **b**, **c** Kinetic binding curves of the top five mAbs to the RBD protein (**b**) and the prefusion S protein (**c**) of SARS-CoV-2. The vertical dashed lines indicate the separation of association and dissociation phases. **d** Epitope binning of 15 mAbs by a BLI-based cross-competition assay. Antibodies grouped into different bins shown in different colors. The top five neutralizing mAbs are shown in red; “+” denotes that the first antibody competes with the second antibody and “−” denotes that the first antibody does not compete with the second antibody. **e** Simultaneous binding of CoV2-06 and CoV2-14 on the sCoV2-RBD protein. **f** Dose-dependent percent neutralization of SARS-CoV-2 by individual CoV2-06, CoV2-14 mAbs, and a cocktail of the two mAbs; *n* = 3 biologically independent cells. **g** Plot of calculated log-scale CI values (*y*-axis) versus fractional effects (*x*-axis). CI value =1 indicates additive effect, <1 means synergism, and >1 indicates antagonism. Error bars indicate SD of triplicates.

We assessed the neutralization potency of the 11 mAbs with NT_7_ <10 μg/ml by a titration assay. Their 50% neutralization titers (NT_50_s) were between 0.15 and 5.2 μg/ml (Fig. 2a and Supplementary Fig. 3a). The top five mAbs (CoV2-06, 09, 14, 16, and 26) had NT_50_ values below 1 μg/ml, with CoV2-06 being the most potent (Fig. 2a). We determined the kinetic binding affinities (*K*_D_) of the 11 mAbs to the RBD with a biolayer interferometry (BLI) assay and the equilibrium binding affinities (EC_50_) with an ELISA titration. The *K*_D_ values were between 1.73 and 20.8 nM (Fig. 2b and Supplementary Fig. 3b). The EC_50_ values were between 0.18 and 7.77 nM (Supplementary Fig. 3c, d). The neutralizing activities (NT_50_) did not correlate with binding affinities to RBD, *K*_D_ values or EC_50_ values (Supplementary Fig. 3e). For the top five mAbs, their apparent affinities (avidities) to the trimeric S protein were between 0.22 and 5.35 nM (Fig. 2c).

After characterizing the binding affinity and neutralizing activity of these mAbs, we sought to identify antibody partners suitable to formulate a cocktail. We selected the 11 potent neutralizing mAbs, together with the two cross-reactive mAbs (CoV2-12 and CoV2-20) and two relatively weak neutralizing mAbs (CoV2-32 and CoV2-33). We performed epitope binning for these selected mAbs, evaluating their ability to compete with each other for binding of RBD. These mAbs delineated five epitope bins, which were designated bin 1 to 5 (Fig. 2d). The top five neutralizing mAbs (CoV2-06, 09, 14, 16, and 26) were in bins 2–4: two in bin 2 (CoV2-06 and 26), two in bin 3 (CoV2-09 and 16), and one in bin 4 (CoV2-14). The two cross-reactive mAbs (CoV2-12 and CoV2-20) were grouped into bin 1. The two weak neutralizing mAbs (CoV2-32 and CoV2-33) and mAb CoV2-29 were grouped into bin 5. Bins 2, 3, and 4 are closely related; antibodies in adjacent bins demonstrated some degree of cross-competition. We selected CoV2-06 (bin2) and CoV2-14 (bin4) for combination studies because they are among the top five neutralizing mAbs and can simultaneously bind to the RBD (Fig. 2d, e). The cocktail combination of CoV2-06 and CoV2-14 showed a synergy in neutralizing SARS-CoV-2 in vitro (Fig. 2f, g). These data suggest that CoV2-06 and CoV2-14 are ideal partners to formulate a cocktail.

### Molecular determinants on the RBD for cocktail mAbs

**Fig. 3:**
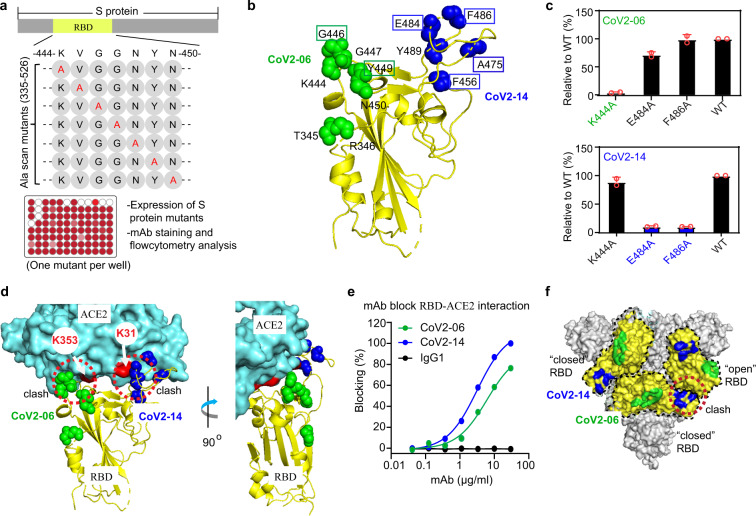
Molecular determinants on the RBD for CoV2-06 and CoV2-14 binding and the mechanism of neutralization. **a** Schematic diagram of the shotgun and high-throughput epitope mapping strategy. Representative alanine scan mutations in the RBD region of S and the critical procedures for mapping are shown. **b** The residues critical for CoV2-06 and CoV2-14 are shown as green and blue spheres, respectively, on a structure of RBD (PDB: 6M0J). The residues that make direct contact with ACE2 are boxed. **c** CoV2-06 or Cov2-14 binding to the sCoV2-RBD proteins with indicated mutations. Error bars indicate SD of duplicates wells. **d** The critical residues for CoV2-06 and CoV2-14 at the interface of RBD-ACE2 complex (PDB: 6M0J). The arrows indicate the K353 and K31 residues in ACE2, which are two virus-binding hotspots. The dashed circles indicate the steric clash of the two mAbs and ACE2 in binding to the RBD. **e** Dose-dependent blocking of RBD binding to ACE2 by CoV2-06 and Cov2-14. **f** The landscape of CoV2-06 and CoV2-14 epitopes on the trimeric S structure (PDB: 6VSB). The RBD in each monomer is outlined and colored in yellow. The CoV2-06 epitope is colored in green and the CoV2-14 epitope in blue. The dashed circle indicates a steric clash of CoV2-14 and an adjacent “open” RBD in binding to a “closed” RBD.

To define the critical RBD residues for binding of CoV2-06 and CoV2-14, we constructed a comprehensive SARS-CoV-2 RBD mutation library using the full-length protein, in which 184 residues of the RBD (between residues 335–526) were individually mutated to alanine, and alanine residues to serine. Each S protein RBD mutant was individually expressed in HEK293-T cells for determining mAb binding (Fig. 3a). The residues T345, R346, K444, G446, G447, Y449, and N450 were identified as critical for CoV2-06 binding. The residues F456, A475, E484, F486, and Y489 were identified as critical for CoV2-14 binding (Fig. 3b and Supplementary Table 1). All of these critical residues except T345 and R346 were in the RBM; six were in direct contact with ACE2 (ref. ^31^) (Fig. 3b). Importantly, none of the critical residues for the two mAbs overlapped. In support of the mapping results, in an ELISA analysis of mAb binding to sCoV2-RBD, the K444A mutation abolished the binding of CoV2-06. Likewise, the E484A or F486A mutations abolished the binding of CoV2-14 (Fig. 3c). The K31 and K353 residues in ACE2 are two virus-binding hotspots in the RBD-ACE2 interface^8^. Interestingly, the CoV2-14 epitope residues E484 and F486 are in direct contact with ACE2 K31 (ref. ^31^) and the CoV2-06 epitope is adjacent to the ACE2 K353 (Fig. 3d). The epitope locations indicate that each of the two mAbs target a different hotspot to block ACE2 binding. In agreement with this result, both CoV2-06 and CoV2-14 inhibited sCoV2-RBD binding to ACE2 in a dose-dependent manner (Fig. 3e). We also visualized the epitope locations on the trimeric S protein to analyze their antibody accessibility. On the trimeric S protein, the CoV2-06 epitope is accessible in both the “open” and “closed” RBD. In contrast, the CoV2-14 epitope is more accessible in the “open” RBD than the “closed” RBD, especially if the “closed” RBD is adjacent to an “open” RBD (Fig. 3f). Antibodies that target both the more accessible and less accessible sites can have high potency^12,32^.

**Fig. 4:**
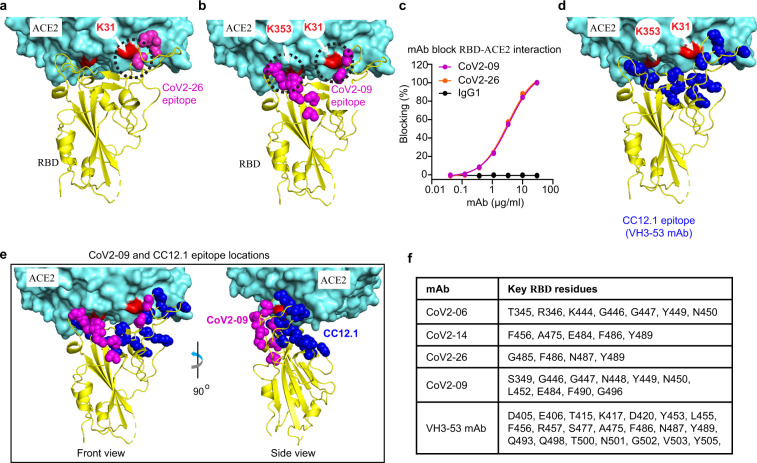
Molecular determinants on the RBD for binding by CoV2-26, CoV2-09, and VH3-53-like antibodies. **a**, **b** The residues critical for CoV2-26 (**a**) and CoV2-09 (**b**) binding are shown as magenta spheres on the RBD–ACE2 complex (PDB: 6M0J). The arrows indicate the K353 and K31 residues in ACE2, which are two virus-binding hotspots. The dashed circles indicate the clash of mAb and ACE2 in binding to the RBD. **c** Dose-dependent blocking of RBD binding to ACE2 by the mAbs. **d** The residues critical for the VH3-53 antibody CC12.1 are shown as blue spheres on the RBD–ACE2 complex (PDB: 6M0J). **e** Comparison of the critical residues for the CoV2-09 and the CC12.1 antibody. **f** The RBD residues critical for binding of the indicated mAbs.

We also mapped the epitopes of CoV2-09, CoV2-16, and CoV2-26 from the top five potent neutralizing mAbs (Fig. 4 and Supplementary Table 1). Interestingly, although CoV2-14 and CoV2-26 bind to similar RBD epitope and shared two critical residues (Figs. 3b and 4a, f), only CoV2-14 could bind to RBD simultaneously with CoV2-06 (Fig. 2d). This suggests that CoV2-26 may have a different approaching angle that is not compatible with simultaneous binding by CoV2-06. Thus, we understand that both non-overlapping epitopes and lack of competition for binding are critical determinants for selecting cocktail mAbs. CoV2-09 and CoV2-16 have the same critical residues (Supplementary Table 1), possibly because they share the same heavy chain (Supplementary Fig. 2a). Therefore, we focused on CoV2-09 for further analysis. The binding epitopes of CoV2-09 and CoV2-26 suggest that they are also ACE2-competing mAbs (Fig. 4a, b). This was validated in a BLI-based competition assay (Fig. 4c). Unlike CoV2-06, CoV2-14, or CoV2-26 mAbs, CoV2-09 had an epitope that is adjacent to both the K353 and K31 hotspots in ACE2 (Fig. 4b), which was similar to the VH3-53-like antibodies^32^ (Fig. 4d). However, the epitopes of CoV2-09 and VH3-53 mAbs were distinct from each other with no overlapping residues (Fig. 4e, f). A number of VH3-53-like antibodies with potent neutralizing activities have been isolated and their epitopes are defined^32^. Due to the promising nature of these antibodies, future studies shall determine whether CoV2-09 and VH3-53-like mAbs can simultaneously bind to RBD and to form an effective cocktail. Collectively, these data elucidated the molecular basis for CoV2-06 and CoV2-14 as effective cocktail mAbs, and also identified other determinants potentially suitable for designing mAb cocktails.

CoV2-12 is a rare mAb that cross-reacts with SARS-CoV-2 and SARS-CoV. We further characterized its binding, neutralization, and epitope (Supplementary Fig. 4 and Supplementary Table 1). CoV2-12 binds to the RBD proteins of the SARS-CoV-2 and SARS-CoV with comparable EC_50_ values (Supplementary Fig. 4a). It also binds to the S proteins of the SARS-CoV-2 and SARS-CoV, but not the MERS-CoV, with comparable avidities (Supplementary Fig. 4b–d). CoV2-12 neutralizes the SARS-CoV-2 with an NT_50_ of 18.17 μg/ml (Supplementary Fig. 4e). These results indicate that CoV2-12 targets a conserved neutralizing epitope. Epitope mapping indicated that CoV2-12 binds an RBD site distal from the RBM (Supplementary Fig. 4f). This result is consistent with its ability to simultaneously bind to RBD with the top five neutralizing mAbs (Fig. 2d). The critical residues identified for CoV2-12 (T385, N388, and F392) are completely conserved between SARS-CoV-2 and SARS-CoV (Supplementary Fig. 4g). Two residues (T385 and F392) overlapped with the contact residues of the cross-reactive mAb CR3022 (ref. ^33^), but no residues overlapped with several other reported cross-reactive mAbs, including VHH-72 (ref. ^18^), S309 (ref. ^19^), and H104 (ref. ^20^). The N388 was a critical residue unique to CoV2-12 (Supplementary Fig. 4f, h). Although the epitopes of CoV2-12 and CR3022 partially overlapped, CoV2-12 exhibited 50% neutralization at 18.17 μg/ml while CR3022 exhibited no neutralization against SARS-CoV-2 at 400 μg/ml^33^. The difference in critical contact residues may be one reason these two mAbs behave differently in neutralizing SARS-CoV-2.

### CoV2-06 and CoV2-14 cocktail prevents neutralization escape of live SARS-CoV-2

**Fig. 5:**
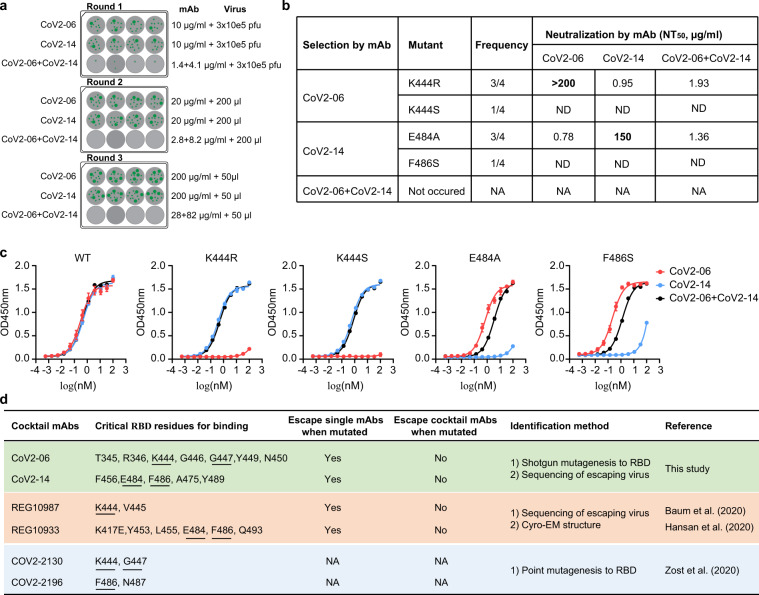
CoV2-06 and CoV2-14 cocktail prevents escape mutation of live SARS-CoV-2. **a** Schematic diagram for the procedures of evaluating SARS-CoV-2 escape mutation under individual or cocktail mAbs. Green dots represent cell clusters expressing the mNeonGreen due to viral infection. **b** The mutated RBD residue, occurring frequency, and mAb neutralization of the mutant viruses. ND not determined, NA not available. **c** ELISA binding curves of indicated mAb to wild-type (WT) or mutant sCoV2-RBD proteins. Data points are mean ± SD of two replicates. **d** Summary of the key residues, the ability to inhibit mutant virus, and the methods of identifying the critical residues for cocktail mAbs in this study and published studies.

We used the authentic live SARS-CoV-2 to evaluate neutralization escape. We passaged the SARS-CoV-2-mNG virus in the presence of CoV2-06, CoV2-14, CoV2-06+CoV2-14 for three rounds (Fig. 5a). We could recover virus in the presence of individual CoV2-06 or CoV2-14 mAbs but not in the presence of the cocktail mAbs (Fig. 5a). We then sequenced the S region of the viruses recovered from the four replicative selections to identify escape mutations. Under CoV2-06 selection, three independently selected viruses had a K444R mutation and one selected virus had a K444S mutation. Under CoV2-14 selection, three independently selected viruses had an E484A mutation and one selected virus had an F486S mutation (Fig. 5b). Outside the RBD, additional mutations in the N-terminal domain (NTD) of S were also observed in some selected viruses. For example, H66R or H66R+R190K were observed under CoV2-06 selection, and N74K was observed under CoV2-06 or CoV2-14 selection. We sought to confirm whether the mutations in the RBD but not the NTD are responsible for resistance. We focused on the most frequent K444R and E484A mutations and constructed two recombinant SARS-CoV-2 viruses with point mutation K444R or E484A. The two mutant viruses were then analyzed for neutralization by individual CoV2-06, CoV2-14 mAbs, and the CoV2-06+CoV2-14 cocktail. The K444R mutant virus could escape the neutralization by CoV2-06 but not CoV2-14; the E484A mutant virus could escape the neutralization by CoV2-14 but not neutralization by CoV2-06 (Fig. 5b). The mAb cocktail maintained neutralization against both the K444R and E484A mutant viruses (Fig. 5b). These results demonstrated that the mAb cocktail of CoV2-06 and CoV2-14 is effective in preventing SARS-CoV-2 escape mutations in vitro.

Next, we sought to further investigate whether the loss of neutralization of single mAbs to the mutant viruses is the result of diminished RBD binding activities. Toward this end, we generated four sCoV2-RBD proteins with individual mutations of K444R, K444S, E484A, or F486S. While CoV2-06 had almost no binding to sCoV2-RBD proteins with K444R or K444S mutations, it maintained binding to sCoV2-RBD proteins with E484A and F486S mutations (Fig. 5c). Similarly, CoV2-14 lost binding to sCoV2-RBD proteins with E484A or F486S mutations but maintained binding to sCoV2-RBD proteins with K444R or E484A mutations (Fig. 5c). The mAb cocktail maintained binding to all the sCoV2-RBD mutant proteins (Fig. 5c). These binding data are in agreement with the epitope mapping findings that CoV2-06 and CoV2-14 target two non-overlapping epitopes. These results indicate that mutations on a single antigenic site do not abolish the binding and neutralization of CoV2-06+CoV2-14 at the same time, which may occur for cocktail mAbs with overlapping epitopes^24^.

Three independent studies, including ours, have identified and characterized cocktail mAbs targeting the RBD^15,16,24^. Figure 4d summarized the RBD residues critical for the cocktail mAbs and escape mutations. Interestingly, mAbs CoV2-06, REG10987, and COV2-2130 shared the same critical residue K444; mAbs CoV2-14, REG10933, and COV2-2196 shared the same critical residue F486. Other shared residues, such as G447 for CoV2-06 and COV2-2130, E484 for CoV2-14 and REG10933, were also observed (Fig. 5d). Both CoV2-06+CoV2-14 and REG10987+REG10933 cocktails were able to prevent viral escape while individual mAbs were not (Fig. 5d). The critical residues on the RBD for these analogous mAb cocktails are key determinants for formulating optimal mAb cocktails against SARS-CoV-2.

### CoV2-06 and CoV2-14 cocktail imposes a strong mutational constraint on the RBD

**Fig. 6:**
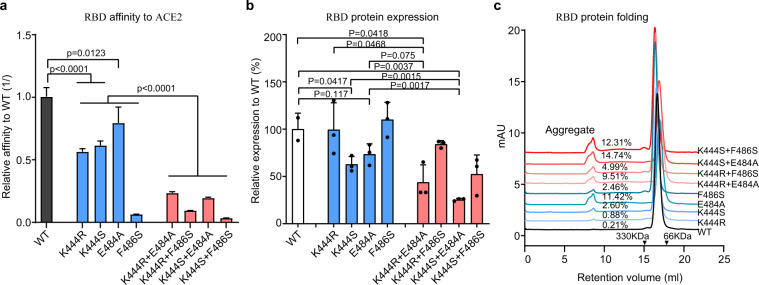
Effects of single-site or double-site mutations on the RBD affinity to ACE2, the expression level, and the folding stability of RBD. **a** The relative binding affinities of the sCoV2-RBD mutant proteins ACE2. The *y*-axis indicates the reversed value of *K*_D_ of mutants/WT. Data are mean ± SD of the *K*_D_ values from fitting of five kinetic curves. Two-tailed Student’s *t*-test. The distribution of data points is not available from the Octet Data Analysis software. **b** The relative expressing levels of the sCoV2-RBD mutant proteins to wild-type (WT) protein. The *y*-axis indicates the value of protein concentration of mutants/WT. Data are mean ± SD of triplicate wells of transfection. Two-tailed Student’s *t*-test. **c** The size-exclusion chromatography (SEC) analysis of purified sCoV2-RBD mutant or wild-type proteins. The retention volume of proteins with indicated molecular weight are shown by arrowheads. The percentages of protein aggregates are shown.

While some RBD mutations are well tolerated, other mutations are deleterious for RBD function and therefore constrained in SARS-CoV-2^22^. We reasoned that simultaneous mutations on the two distinct binding sites of CoV2-06 and CoV2-14, which are required for virus to escape neutralization by the cocktail, would be more constrained than mutations on the binding sites of individual mAbs. To test this hypothesis, we generated eight sCoV2-RBD mutant proteins, four with individual mutations of single binding sites (K444R, K444S, E484A, and F486S) and four with simultaneous mutations of both binding sites (K444R+E484A, K444R+F486S, K444S+E484A, and K444S+F486S) (Supplementary Fig. 5a). These single-site or double-site RBD mutants were analyzed for their affinity to ACE2 (Fig. 6a and Supplementary Fig. 5b–j), protein expression (Fig. 6b), and folding stability (Fig. 6c). Single-site mutations of K444R, K444S, E484A, and F486S reduced the sCoV2RBD/ACE2 binding affinities to 56%, 61%, 79%, and 6% of the wild-type (WT), respectively. In comparison, double-site mutations of K444R+E484A, K444R+F486A, K444S+E484A, and K444S+F486S further reduced the sCoV2-RBD/ACE2 binding affinities to 23%, 9%, 19%, and 3% of the WT, respectively (Fig. 6a and Supplementary Fig. 5b–j). Similarly, while single-site mutations altered the RBD expression to 69–110.1% of the WT, double-site mutations reduced the expression to 25.5–84.2% of the WT (Fig. 6b). The size exclusion chromatography (SEC) analysis showed protein aggregates of 0.88–11.42% and 4.99–14.74% for RBD with single-site mutations and double-site mutations, as compared to only 0.21% of aggregates for WT RBD (Fig. 6c). These data indicate that double mutations at both the CoV2-06 and CoV2-14 epitope sites attenuated the receptor-binding affinity and stability of the RBD more than that of the single-site mutations, suggesting that such double-site mutations would have deleterious effects on viral fitness.

### No evidence for the occurrence of virus variants with double-site mutation

**Fig. 7:**
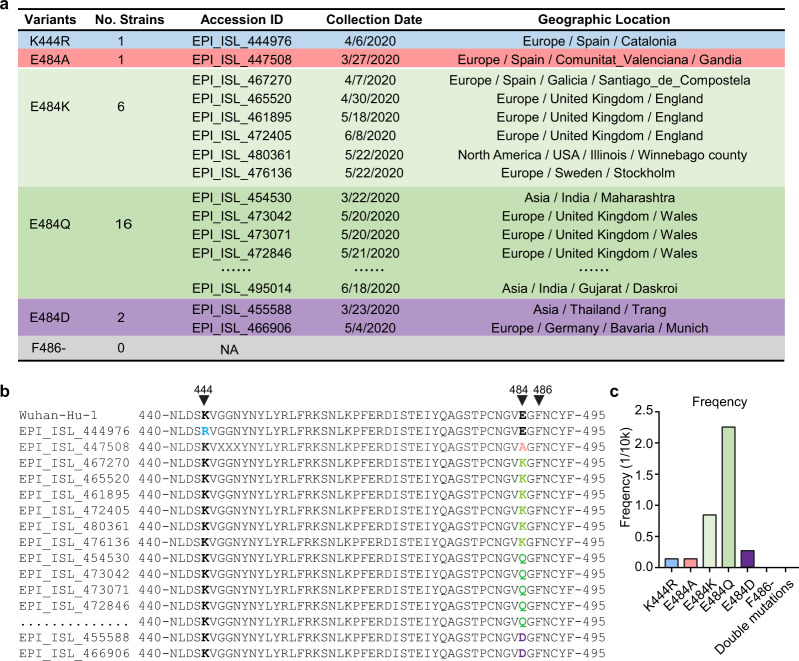
Sequence analysis of SARS-CoV-2 isolates with natural mutations at the K444, E484, or F486 sites of the RBD. **a** Summary of total numbers, accession ID, collection date, and geographic locations for the clinical SARS-CoV-2 isolates with indicated mutations. A total of 70,943 viral genome sequences were queried from GISAID and analyzed. **b** Alignment of the RBD sequences of the mutant viruses with the reference Wuhan-Hu-1 strain. **c** Frequency of the virus variants with single mutations of the K444, E484, and F486 residues, or simultaneous mutations of K444+E484 or K444+F486 residues in the total analyzed viral sequences.

To gain insights of RBD mutations in naturally occurred virus variants during the global transmission, we analyzed 70,934 publicly available viral genome sequences (as of July 23, 2020) (Fig. 7). Although the frequency was very low, 26 clinical isolates with RBD polymorphisms analog to the mAb-escaping mutants were identified. Among these isolates, one had the K444R mutation, one had the E484A mutation, six had the E484K mutation, 16 had the E484Q mutation, and two had the E484D mutation. No polymorphism at F486 was found (Fig. 7a). It is likely that these virus variants occurred as a result of selection by the epitope-directed neutralizing antibodies in COVID-19 patients^15,24^. However, the low frequency of occurrence indicates that these virus variants may have compromised epidemiologic fitness during transmission. More importantly, alignment of the RBD regions of these 26 isolates demonstrated no occurrence of virus variants with simultaneous mutations on the K444 and E484 sites, or the K444 and F486 sites, to the date of analysis (Fig. 7b, c). These results suggest that virus variants with simultaneous mutations at both CoV2-06 and CoV2-14 epitopes either have not occurred or occurred at an extremely low frequency that is beyond epidemiologic monitoring.

### Antibody protection against SARS-CoV-2 infection in mice

**Fig. 8:**
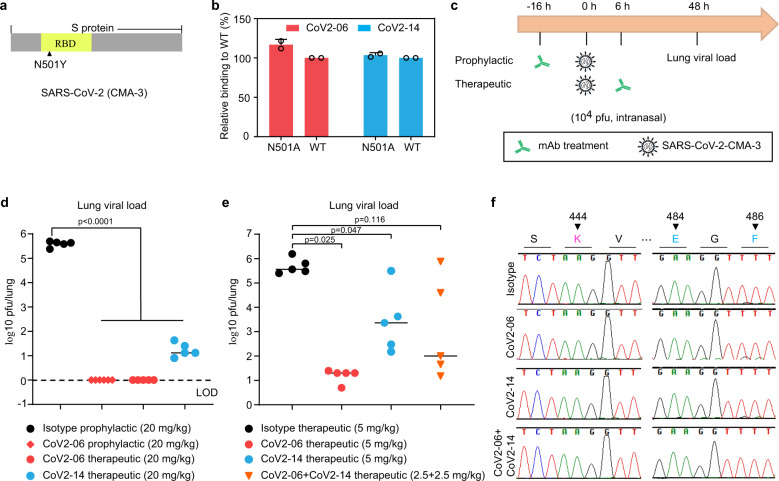
Antibody protection of SARS-CoV-2 infection in mice. **a** A diagram showing the N501Y adapted mutation in the S protein RBD of the SARS-CoV-2 mouse-adapted strain (CMA-3). **b** ELISA binding of CoV2-06 and CoV2-14 to the WT sCoV2-RBD or the N501A mutant. Error bars indicate SD of duplicates wells. **c** Schematic diagram of prophylactic or therapeutic evaluations of the antibodies. **d** The infectious viral load in the lung of CoV-06- or CoV2-14-treated mice compared to that of isotype IgG1-treated mice. The dashed line indicates the limit of detection (LOD) of the assay. **e** The infectious viral load in the lung of mice with indicated treatment. The median levels of the lung viral load were shown as solid lines. *N* = 5 mice. Ordinary one-way ANOVA with Sidak’s multiple comparison test. **f** Representative sequencing results of the RBD regions of the viruses harvested from each treatment groups. The amino acid residues critical for antibody binding were indicated by triangles.

We evaluated the protective effects of CoV2-06 and CoV2-14 individually and in combination in a mouse model of infection with a mouse-adapted virus strain (CMA-3). This virus has a N501Y adaptive mutation in the RBD of the S region to facilitate mouse infection (Fig. 8a). Utilization of mouse-adapted virus in evaluation of vaccine efficacy and antibody protection in mice had been demonstrated elsewhere^34–36^. The N501 was not a critical RBD residue for CoV2-06 or CoV2-14. We confirmed that mutation of N501 did not reduce RBD binding by the two antibodies (Fig. 8b). The CoV2-06 mAb is the most potent neutralizing mAb in this study. Its neutralization activity was independently validated by using SARS-CoV-2 S pseudovirus (Supplementary Fig. 6a) and the SARS-CoV-2 clinical isolate (USA/WA1/2020) (Supplementary Fig. 6b, c). To evaluate antibody protection in vivo, mice were given an intraperitoneal injection of CoV2-06 at 16 h before or 6 h after intranasal challenge with 10^4^ plaque-forming unit (pfu) of the CMA-3 virus. Two days post infection, the lung tissues were harvested and the viral loads were measured (Fig. 8c). For both the prophylactic and therapeutic treatments, CoV2-06 at 20 mg/kg reduced lung viral load to undetectable level. Therapeutic treatment with CoV2-14 at 20 mg/kg reduced lung viral load by 4–5 log10 fold (Fig. 8d). We further demonstrated that therapeutic treatments with CoV2-06 and CoV2-14 individually and in combination conferred protection in the same model. The cocktail was less effective than CoV2-06 alone possibly due to the lower dose (5 mg/kg) used (Fig. 8e). Treatment with the sub-optimal dose allow virus harvesting in the lung for determination whether resistant mutations occurred. For both CoV2-06 and CoV2-14 monotherapy groups and the cocktail antibody treatment, no mutation on key epitope residues was observed (Fig. 8f). These data demonstrate effective antibody protections against SARS-CoV-2 in mice.

## Discussion

We identified the molecular determinants on the RBD that are optimal for selecting effective mAb cocktails against SARS-CoV-2. We also revealed the mechanism by which a mAb cocktail prevents escape mutations using live SARS-CoV-2 in cell culture. A mAb cocktail (REG10987+REG10933) has entered phase 2/3 clinical trials (NCT04425629, NCT04452318). Using the VSV-SARS-CoV-2 S recombinant virus, neutralization escape had been evaluated for the REG10987+REG10933 cocktail^24^. Interestingly, we independently identified a mAb cocktail (CoV2-06+CoV2-14) that shares similar binding epitopes with the REG10987+REG10933 cocktail. As VSV and SARS-CoV-2 are different, we used the authentic SARS-CoV-2 to evaluate neutralization escape. Although different viral systems were used, both studies demonstrated that only the mAb cocktail, not individual mAbs, can prevent escape mutations^24^. Indeed, SARS-CoV-2 escapes from individual mAb inhibition rapidly, within 2–3 passages, regardless of mAb neutralization potency^24^. Amino acid K444 is a critical RBD residue for both CoV2-06 and REG10987. Amino acids E484 and F486 are critical RBD residues for both CoV2-14 and REG10933. Functional analysis validated that mutations of these residues are responsible for viral escape from the individual mAbs. Amino acid K444 is also a critical epitope residue for other SARS-CoV-2 neutralizing mAbs, including P2B-2F6 and S309 (refs. ^12,19^); E484 is a critical residue for P2B-2F6 (ref. ^12^); and F486 is a critical residue for VH3-53-like mAbs^32^. We show that single-site mutations of these residues compromised the RBD on its affinity for ACE2 and the folding stability slightly, but double-site mutations attenuated the fitness of RBD dramatically. These results are consistent with evidence showing no natural occurrence of virus variants with simultaneous mutations of the binding residues for the two mAbs. Our findings provide mechanistic insights into how such cocktails prevent viral escape. It is important in future studies to evaluate whether antibody cocktails can prevent viral escape in vivo. In this particular experiment, we did not see resistance-related mutation in single antibody or cocktail antibody treated mice. This perhaps because the frequency of mutant virus, if any emerged, was extremely low. Nevertheless, we need to mention that virus escape of neutralizing antibodies may be different in mouse and human systems. Monitoring the dynamic changes of these key mutation sites in clinical studies of antibody monotherapy or cocktail therapy and characterizing their impact on viral pathogenesis will fill this important knowledge gap.

To date, three studies, including ours, identified anti-SARS-CoV-2 mAb cocktails and characterized their epitopes^15,16,24^. Surprisingly, these studies independently discovered cocktail mAbs that have similar epitope combinations and share several key amino acid residues. Because the two epitopes are located on different RBM ridges that are well separated (Fig. 3b)^8^, they are more likely to accommodate two mAbs simultaneously. Binding on non-overlapping epitopes does not necessarily allow simultaneous binding of two mAbs, which is the case for CoV2-26 and CoV2-06. Although CoV2-26 binds to similar epitope and shares key residues with CoV2-14, it is a less than ideal partner than CoV2-14 for combination with CoV2-06 due to the RBD binding competition. This suggests that favorable approaching angles are also important for optimal mAb cocktails. Further structural characterization of the RBD in complex with these mAbs will provide additional insights to understand optimal mAb cocktails. Epitope combinations other than that of CoV2-06 and CoV2-14 might also be attractive to select mAb cocktails. Using CoV2-09, we defined a previously unreported neutralizing epitope. Interestingly, this epitope partially overlapped with epitopes of CoV2-06 and CoV2-14 but not with the epitope of VH3-53-like antibodies (Fig. 4f). Indeed, the epitopes of CoV2-09 and VH3-53-like antibodies are located at two different RBD patches that comprise the ACE2 interface and do not share binding residues (Fig. 4e, f). These epitopes may permit simultaneous binding of the RBD by CoV2-09 and VH3-53-like antibodies. A number of VH3-53-like antibodies have been evaluated as monotherapies^17,29^. However, it is not known if they can prevent viral escape. Therefore, future efforts may focus on validating the combination of CoV2-09 and VH3-53-like mAbs as cocktails to prevent viral escape.

In addition to identifying cocktail mAbs and determining their epitopes, we identified a SARS-CoV-2 neutralizing mAb (CoV2-12) with cross-reactivity to SARS-CoV. Epitope mapping identified three critical RBD residues, which are not overlapped with the epitopes of a number of reported cross-reactive mAbs, including VHH-72, S309, and H104 (refs. ^18–20^). Two of the CoV2-12 epitope residues overlap the epitope of CR3022. However, only CoV2-12 exhibits neutralizing activity against SARS-CoV-2. This suggests that other non-overlapped residues, such as N388, might be important for CoV2-12 to exhibit neutralization. Since the epitope residues of CoV2-12 are located in the RBD core region where amino acid mutations are usually deleterious for RBD expression and folding^22^, certain key epitope residues for CoV2-12 might not be revealed by the RBD mutation library approach that we used. Further structural analysis will provide additional information on this conserved epitope. By focusing on this epitope, future efforts of antibody selection may generate potent and broadly neutralizing mAbs against SARS-CoV-2 and SARS-CoV.

The neutralizing epitopes identified in our study will also be useful for assessing vaccine-elicited antibody responses. To avoid potential escape mutations, it is critical for the vaccines to elicit neutralizing antibodies targeting diverse epitopes. Indeed, antibody responses to the two epitopes of CoV2-06 and CoV2-14 are subdominant in some subjects^15^, while antibody response to the VH3-53-like antibody epitope is shared in many subjects^32^. In addition, the knowledge on these two epitopes can facilitate the design of vaccines that can elicit dominant antibody responses to these epitopes so that viral escape mutations can be reduced. The knowledge on CoV2-12 epitope is useful for the design of vaccines with potential to elicit more broadly neutralizing antibodies.

In summary, we report the molecular determinants and mechanism for a mAb cocktail that prevents SARS-CoV-2 viral escape mutations. We also identified an epitope combination potentially suitable for the design of other cocktail mAbs, as well as a conserved epitope for selecting cross-reactive neutralizing mAbs. Our study is informative for the evaluation of the clinical-stage cocktail mAbs, benefits further selection of other cocktail antibodies against SARS-CoV-2, and aids the assessment of vaccines. Finally, the mAbs we isolated hold promise for further development as antibody therapies for COVID-19.

## Methods

### Expression and purification of RBD proteins

The RBD (R319-F541) of the *spike* of SARS-CoV-2 (Gene Bank: MN908947.3) and the RBD (R306-F527) of the *spike* of SARS-CoV (Gene Bank: AY278489.2) were fused with a human IgG1 Fc fragment and inserted into expression vectors. The constructed plasmids were transiently transfected into Expi293F cells for protein expression. After 6 days, the culture supernatants were harvested, and the proteins were affinity purified using Protein A resin. The proteins were named as sCoV2-RBD and sCoV-RBD, respectively. The protein purities were assessed by SDS-PAGE, and their binding activities to ACE2 were tested by a BLI assay. The sCoV2-RBD proteins with mutations were generated by the same method. For comparison of protein expressing level, plasmids expressing WT or mutant sCoV2-RBD proteins were used to transfect Expi293F cells in triplicates; after 4 days of transfection, the cell supernatant were harvested and the protein concentrations were quantitated on the Octet RED96 system.

### Phage library panning and selection of mAbs targeting the RBD

The sCoV2-RBD protein was used for antibody selection by panning a large human scFv phage display antibody library (containing ~10^12^ antibodies). The library was constructed in house from the cDNA extracted from the PBMCs and tonsils of multiple donors^37^. In each round of phage panning, 50 µg of sCoV2-RBD was coated on a MaxiSorp immune tube and blocked by 8% milk. The phages were pre-blocked by 8% milk and then pre-absorbed by an Fc antigen for deselection. The pre-blocked and deselected phages were then incubated with the antigen pre-coated on the immune tube. After washing with PBST and phosphate-buffered saline (PBS), the phages were eluted by triethylamine (TEA). The eluates were titered and infected *Escherichia coli* TG1 for phage amplification for next round of panning. Similar procedures were performed in round 2 of panning with increased washing stringency. After two rounds of panning, the phage eluates were used to infect *E. coli* TG1 to grow single colonies for picking by QPix420 system (Molecule Devices) and for phage preparation. Individual phage clones were tested for ELISA binding to sCoV2-RBD, sCoV-RBD, and Fc control. The HRP-conjugated mouse-anti-M13 antibody (Santa Cruz, #sc-53004 HRP) was used for detection of antigen-bound phages. The sCoV2-RBD-positive clones were sequenced for their scFv sequences to obtain unique phage binders.

### DNA sequencing, germline gene analysis of antibodies

The phagemids of sCoV2-RBD-positive phage clones were prepared by QIAGEN BioRobot 8000 and sequenced in the scFv region using a specific primer (Supplementary Table 2). Online IMGT/V-QUEST analysis of antibody sequences resulted in the report of the germline genes origins, the V-region identities, and the length of CDR for the VH and Vκ/λ.

### Expression and purification of antibodies

After sequence analysis, the VH and Vκ/λ were PCR amplified and inserted into the IgG1 heavy chain and corresponding light chain backbones. The plasmids were transfected into Expi293F cells and cultured for 7 days. The supernatants were collected, and antibodies were purified using Protein A resin. All the antibody preparations were reconstituted in PBS buffer for the studies.

### Neutralization assay with live SARS-CoV-2

The neutralization assay for the 30 antibodies at 10 µg/ml, neutralization titration assay for the 11 neutralizing antibodies, and the synergistic neutralization assay were performed using the SARS-CoV-2-mNG virus generated before^30^. A total of 1.5 × 10^4^ Vero cells in phenol red-free culture medium were plated into each well of a black transparent flat-bottom 96-well plate (Greiner Bio-One; Cat# 655090). On the next day, antibodies (single dilution or twofold serial dilutions) were mixed with an equal volume of SARS-CoV-2-mNG virus (MOI = 0.5) After 1 h incubation at 37 °C, the antibody–virus complexes were inoculated into the 96-well plate containing confluent Vero cells. The infections were performed in duplicates or triplicates. At 20 h post-infection, nuclei were stained by the addition of Hoechst 33342 (Thermo Fisher Scientific) to a final concentration of 10 nM. Fluorescent images were acquired using a Cytation 7 multi-mode reader (BioTek). Total cells (in blue) and mNG-positive cells (in green) were counted, and the infection rate was calculated. The relative infection rates were calculated by normalizing the infection rate of each well to that of control wells (no antibody treatment). The relative infection rate versus the log10 value of the concentration was plotted, and the 50% neutralization concentration (NT_50_) was obtained by using a four-parameter logistic regression model from the GraphPad Prism 8 software.

The activity for the most potent neutralizing antibody CoV2-06 was validated in another live virus assay using WT SARS-CoV-2 (Isolate USA/WA1/2020) in Vero-E6 cells. The antibody was subjected to twofold dilutions in DMEM 2% FBS from 12.5 µg/ml to 0.048 µg/ml, and mixed with 10 TCID_50_s of SARS-CoV-2 in 96-well plates. Eight replicative wells were set for each antibody concentration. After incubation for 1 h at 37 °C, the mixtures were added to 6000 Vero cells for incubation. After 6 days, the cytopathic effect (CPE) of cells in each well was visually checked under microscopy and the percentages of wells showing CPE were recorded.

### Neutralization synergy analysis

The Chou-Talalay method was used to analyze the cooperation of antibody in neutralization^38^. The CoV2-06 and CoV2-14 were combined (mass ratio of CoV2-06 to CoV2-14 is 1:3) based on their NT_50_ values. Then, antibody alone or in mixture were twofold diluted to cover multiple doses above and below NT_50_s. The neutralization percentages at each dose were entered as fraction affected (Fa, ranging from 0.01 to 0.99) into the CompuSyn software (http://www.combosyn.com/index.html). The dose–effect curves were generated, and combination index (CI) at ED_50_ (50% effective dose), ED_75_, ED_90_, and ED_95_ were calculated based on the Fa–CI plots. CI <1, synergism; CI = 1, additive effect; CI >1, antagonism.

### SARS-CoV-2 S pseudovirus neutralization assay

For preparation of the pseudovirus, the SARS-CoV-2 S expressing 293T cells were infected with VSV-G pseudotyped VSV_Δ_G-RFP—a replication-defective virus encoding a red fluorescent protein reporter in the place of the VSV G glycoprotein. Vero E6 cells stably expressing TMPRSS2 were seeded in 100 μl at 2.5 × 10^4^ cells/well in a 96-well collagen-coated plate. The next day, twofold serially diluted antibody at a starting concentration of 10 μg/ml was mixed with VSV_Δ_G-RFP SARS-CoV-2 pseudotype virus (~150 focus forming units/well) and incubated for 1 h at 37 °C. Also included in this mixture to neutralize any potential VSV-G carryover virus was 8G5F11, a mouse anti-VSV Indiana G, at a concentration of 100 ng/ml (Absolute Antibody, Boston, MA). The antibody–virus mixture was then used to replace the media on VeroE6 TMPRSS2 cells; 20 h post infection, the cells were washed and fixed with 4% paraformaldehyde before visualization on an S6 FluoroSpot Analyzer (CTL, Shaker Heights OH). Individual infected foci were enumerated and the values compared to control wells without antibody.

### ELISA titration of mAb binding and fitting of EC_50_

Corning high-binding assay plates were coated with recombinant sCoV2-RBD or sCoV-RBD protein (1 μg/ml) at 4 °C overnight and blocked with 5% skim milk at 37 °C for 2 h. Serially diluted antibodies were added at a volume of 100 μl per well for incubation at 37 °C for 2 h. The anti-human IgG Fab2 HRP-conjugated antibody was diluted 1:5000 and added at a volume of 100 μl per well for incubation at 37 °C for 1 h. The plates were washed 3–5 times with PBST (0.05% Tween-20) between incubation steps. TMB substrate was added 100 μl per well for color development for 3 min and 2 M H_2_SO4 was added 50 μl per well to stop the reaction. The OD 450 nm was read by a SpectraMax microplate reader. The data points were plotted by GraphPad Prism8, and the EC_50_ values were calculated using a three-parameter nonlinear model.

### BLI measurement of affinity

Antibody affinity was measured on Pall ForteBio Octet RED96 system. Recombinant antibodies (20 μg/ml) was loaded onto the Protein A biosensors for 300 s. Following 10 s of baseline in kinetics buffer, the loaded biosensors were dipped into serially diluted (0.14–300 nM) RBD protein (Sino Biological, Cat: 40592-V08B) or the previously generated S protein for 200 s to record association kinetics^5^. The sensors were then dipped into a kinetic buffer for 400 s to record dissociation kinetics. Kinetic buffer without antigen was set to correct the background. The Octet Data Acquisition 9.0 was used to collect affinity data. For fitting of *K*_D_ value, Octet Data Analysis software V11.1 was used to fit the curve by a 1:1 binding model and use the global fitting method. Similarly, binding affinities to ACE2 by WT sCoV2-RBD protein, mutant sCoV2-RBD proteins, and sCoV-RBD protein were measured.

### Epitope binning of antibodies

Epitope binning was performed on the octet RED96 system using a sandwich format. Briefly, individual antibodies (first antibodies) were diluted to 50 μg/ml and loaded onto Protein A biosensors. After blocking with 200 μg/ml of an irrelevant IgG1, the sensors were dipped into 15 μg/ml of His tagged sCoV2-RBD to capture the antigen. The sensors with antibody–antigen complex were then incubated with the rest antibodies (second antibodies) pairwise in each round of binning. In each round of binning, an isotype IgG1 was used as a control. A total of 15 × 15 sets of antibody binning were performed to obtain the full profile of antibody epitope bins. For data analysis, if a second antibody still binds the RBD pre-captured by a first antibody, the first antibody was defined as competitive with the second antibody; if a second antibody did not bind the RBD pre-captured by a second antibody, the first antibody was defined as non-competitive with the second antibody. The antibody pairs with competition were grouped and defined as the same bin.

### Epitope mapping of antibodies

Epitope mapping was performed using a SARS-CoV-2 (strain Wuhan-Hu-1, QHD43416.1) S protein RBD shotgun mutagenesis mutation library^39^. A full-length expression construct for S protein, where 184 residues of the RBD (between residues 335–526) were individually mutated to alanine, and alanine residues to serine. Mutations were confirmed by DNA sequencing, and clones arrayed in a 384-well plate, one mutant per well. Binding of mAbs to each mutant clone in the alanine scanning library was determined, in duplicate, by high-throughput flow cytometry. Each S protein mutant was transfected into HEK-293T cells and allowed to express for 22 h. Cells were fixed in 4% (v/v) paraformaldehyde (Electron Microscopy Sciences), and permeabilized with 0.1% (w/v) saponin (Sigma-Aldrich) in PBS plus calcium and magnesium (PBS++) before incubation with mAbs diluted in PBS++, 10% normal goat serum (Sigma), and 0.1% saponin. The mAb screening concentrations were determined using an independent immunofluorescence titration curve against cells expressing WT S protein to ensure that signals were within the linear range of detection. Antibodies were detected using 3.75 μg/mL of AlexaFluor488-conjugated secondary antibody in 10% normal goat serum with 0.1% saponin. Cells were washed three times with PBS++/0.1% saponin followed by two washes in PBS and mean cellular fluorescence was detected using a high-throughput Intellicyte iQue flow cytometer (Sartorius). Antibody reactivity against each mutant S protein clone was calculated relative to WT S protein reactivity by subtracting the signal from mock-transfected controls and normalizing to the signal from WT S-transfected controls. Mutations within clones were identified as critical to the mAb epitope if they did not support reactivity of the test mAb, but supported reactivity of other SARS-CoV-2 antibodies. This counter-screen strategy facilitates the exclusion of S mutants that are locally misfolded or have an expression defect. Validated critical residues represent amino acids whose side chains make the highest energetic contributions to the mAb–epitope interaction.

For further validations of some of the critical residues identified, the sCoV2-RBD proteins with specific point mutations were generated to test antibody reactivity. Briefly, the WT and mutant sCoV2-RBD were coated on ELISA plates and tested for binding for all the mAbs at 3 μg/ml. HRP-conjugated goat-anti-human IgG1 Fc was used as a positive control and to normalize the OD450nm values among the mutant and WT sCoV2-RBD proteins. The relative binding of each mutant to WT was calculated as (%) = OD450 nm of mutant protein/ OD450 nm of wild-type protein × 100%.

### Octet-based assay for antibody blocking of RBD and ACE2 interaction

The purified antibodies were tested for their blocking activities against sCoV2-RBD binding to ACE2 on the Octet RED96 system. The sCoV2-RBD (5 μg/ml) was captured on the Protein A biosensors for 300 s. After capture, the biosensors were blocked by 200 μg/ml of Fc protein and then dipped into serial diluted antibody solutions (0–30 μg/ml) for 200 s, and then into ACE2 solution (5 μg/ml) for 200 s. Between each incubation step, there were 10 s of baseline steps. The binding responses were recorded for all incubation steps. To calculate the percent of blocking, the responses of ACE2 binding were first normalized to the beginning point and then normalized against the smallest response value for each antibody set. The percent of blocking was calculated as blocking (%) = (Normalized response of buffer – Normalized response of mAb) / OD450nm Normalized response of buffer × 100%.

### Virus escape from neutralizing antibodies

The SARS-CoV-2-mNG virus and Vero E6 cells were used to select neutralization-escape mutant under individual CoV2-06, CoV2-14, or CoV2-06+CoV2-14 for three rounds. Each selection was performed in four replicative wells in a 12-well format. For the first round of selection, 3 × 10^5^ cells were seeded 1 day prior to infection. On the next day, 6 × 10^5^ pfu of virus was pre-incubated with CoV2-06 (10 μg/ml), CoV2-14 (10 μg/ml), or CoV2-06 (1.4 μg/ml) + CoV2-14 (4.1 μg/ml) and the mixtures were added to cells for incubation for 3 days. The supernatants were harvested as round 1 (R1) virus. For the second round of selection, 200 μl of R1 virus was pre-incubated with CoV2-06 (20 μg/ml), CoV2-14 (20 μg/ml), or CoV2-06 (2.8 μg/ml) + CoV2-14 (8.2 μg/ml) and added to cells for incubation for 2–4 days to generate the R2 virus. For the third round of selection, 50 μl of R2 virus was pre-incubated with CoV2-06 (200 μg/ml), CoV2-14 (200 μg/ml), or CoV2-06 (14 μg/ml) + CoV2-14 (41 μg/ml) and added to cells for incubation for 2–4 days to generate the R3 virus. The expressions of mNG were monitored at each round for indication of infection. After three rounds of selections, the R3 virus for each group was Sanger sequenced of the S region using specific primers (Supplementary Table 2). Antibody neutralizations against the mutant virus were performed as described above.

### SEC analysis

The SEC analysis of WT and mutant sCoV2-RBD proteins were performed on the ÄKTA pure system with the Superpose 6 increase 10/300GL column in PBS buffer. All the proteins preparations were centrifuged at 13,800×*g* for 5 min to remove visible aggregates and 100 μg of proteins were used for each loading. The collagen I, IgG, and BSA were used as molecular markers to indicate the retention volumes. The UNICORN 7.0 software was used to analyze and export the data of each curve.

### Bioinformatics analysis of the RBD sequences of SARS-CoV-2 clinical isolates

As of July 23, 2020, 70,934 human SARS-CoV-2 genomics sequences and information were collected from GISAID. The viral genomes were downloaded and used as query to search against the reference sequence of the RBD (Gene Bank: MN908947.3) via blastx. The RBD regions of the download viral genomes were subsequently gathered via “blastdbcmd” option in ncbi-blast-2.2.30 (ftp://ftp.ncbi.nlm.nih.gov/blast/executables/blast+/2.2.30/). Those coding nucleotides were subsequently translated via standalone version of Orf-Predictor^40^. The predicted protein sequences were aligned and variants were determined via standalone version of Clustal Omega^41^.

### Animal studies

This study was carried out in accordance with the recommendations for care and use of animals by the Office of Laboratory Animal Welfare, National Institutes of Health. The Institutional Animal Care and Use Committee (IACUC) of University of Texas Medical Branch (UTMB) approved the animal studies under protocol 1802011. Ten- to twelve-week-old female BALB/c mice were purchased from Charles River Laboratories and maintained in Sealsafe^TM^ HEPA-filtered air in/out units. A mouse-adapted virus was generated based on a previously reported study. Animals were anesthetized with isoflurane and infected intranasally (IN) with 10^4^ pfu of mouse-adapted SARS-CoV-2 in 50 μl of PBS. Antibodies were intraperitoneally injected (20 mg/kg or 5 mg/kg) at 16 h before or 6 h after viral infection. As a control group, mice were injected with an IgG1 before or after viral infection. Two days after infection, lung samples of infected mice were harvested and homogenized in 1 ml PBS for analysis of infectious virus by plaque assay. The virus harvested from each mice in different antibody treatment (5 mg/kg) groups were individually sequenced of the RBD region using specific primers (Supplementary Table 2).

### Statistical analysis

The statistics for ELISA binding, virus neutralization, and receptor blocking and protein expression levels were calculated with Graphpad Prism 8. The statistics for antibody affinities to the RBD and the RBD to ACE2 were reported with the ForteBio’s data analysis software. The combination effects of antibody cocktail were analyzed using the CompuSyn program.

### Reporting summary

Further information on research design is available in the Nature Research Reporting Summary linked to this article.

## Supplementary information

Supplementary Information

Reporting Summary

## Data Availability

The sequences of SARS-CoV-2 viruses were from the GISAID database (https://www.gisaid.org/). The accession codes and web links of Spike sequences from NCBI gene bank are: MN908947.3; AY278489.2. Source data are provided with this paper.

## Source data

Source Data
