# Author Correction: Molecular determinants and mechanism for antibody cocktail preventing SARS-CoV-2 escape

**DOI:** 10.1038/s41467-021-24440-x

**Published:** 2021-07-01

**Authors:** Zhiqiang Ku, Xuping Xie, Edgar Davidson, Xiaohua Ye, Hang Su, Vineet D. Menachery, Yize Li, Zihao Yuan, Xianwen Zhang, Antonio E. Muruato, Ariadna Grinyo i Escuer, Breanna Tyrell, Kyle Doolan, Benjamin J. Doranz, Daniel Wrapp, Paul F. Bates, Jason S. McLellan, Susan R. Weiss, Ningyan Zhang, Pei-Yong Shi, Zhiqiang An

**Affiliations:** 1 Texas Therapeutics Institute, Brown Foundation Institute of Molecular Medicine, University of Texas Health Science Center at Houston, Houston, TX 77030 USA; 2 Department of Biochemistry and Molecular Biology, Institute for Human Infection and Immunity, Sealy Institute for Vaccine Sciences, Sealy Center for Structural Biology & Molecular Biophysics, Department of Pharmacology & Toxicology, University of Texas Medical Branch, Galveston, TX 77555 USA; 3 Integral Molecular, Philadelphia, Pennsylvania, PA 19104 USA; 4 Department of Microbiology & Immunology, University of Texas Medical Branch, Galveston, TX 77555 USA; 5 Department of Microbiology, Perelman School of Medicine, University of Pennsylvania, Philadelphia, PA 19104 USA; 6 Department of Molecular Biosciences, University of Texas at Austin, Austin, TX 78712 USA

**Keywords:** Antibodies, SARS-CoV-2

Correction to: *Nature Communications* 10.1038/s41467-020-20789-7, published online 20 January 2021.

The original version of this Article contained an error in the Acknowledgements, which incorrectly listed the NIH grant ‘AI142759’. This has been corrected in both the PDF and HTML versions of the Article.

