# Supplementary material for: Molecular determinants and mechanism for antibody cocktail preventing SARS-CoV-2 escape
Source: Nat Commun. 2021 Jan 20;12:469. doi: 10.1038/s41467-020-20789-7 (PMC7817669; doi:10.1038/s41467-020-20789-7)
Supplement: Supplementary file 1 — Supplementary Information [file 41467_2020_20789_MOESM1_ESM.pdf]

Supplementary Figures and Tables

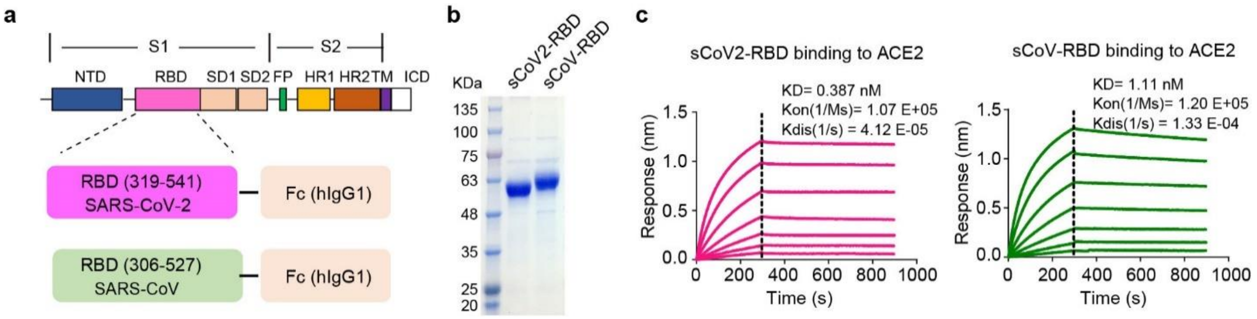

Supplementary Fig. 1. Expression and characterization of the RBD proteins. a Structures of the SARS-CoV-2 spike protein and the RBD-Fc fusion proteins. NTD: N-terminal domain, RBD: receptor-binding domain, SD1: subdomain 1, SD2: subdomain 2, FP: fusion peptide, HR1: heptad repeat 1, HR2: heptad repeat 2, TM: transmembrane region, IC: intracellular domain. **b** SDS-PAGE of purified sCoV2-RBD and sCoV-RBD proteins. **c** Kinetic binding curves of the sCoV2-RBD and the sCoV-RBD to ACE2.

a

mAb	VH					Vκ/λ			
	V-GENE	J-GENE	D-GENE	Identity (%)	Length of CDR [1, 2, 3]	V-GENE	J-GENE	Identity (%)	Length of CDR [1, 2, 3]
★ CoV2-01	IGHV1-2*02 F	IGHJ4*02 F	IGHD3-16*01 F	96.53	[8.8.20]	IGKV1-39*01 F	IGKJ5*01 F	97.49	[6.3.9]
CoV2-02	IGHV1-2*02 F	IGHJ4*02 F	IGHD3-16*01 F	96.88	[8.8.20]	IGKV1-39*01 F	IGKJ4*01 F	94.62	[6.3.9]
CoV2-03	IGHV1-2*02 F	IGHJ4*02 F	IGHD3-16*01 F	96.53	[8.8.20]	IGKV1-39*01 F	IGKJ1*01 F	99.64	[6.3.9]
CoV2-04	IGHV4-34*01 F	IGHJ5*02 F	IGHD6-13*01 F	92.28	[8.7.18]	IGKV1-39*01 F	IGKJ4*01 F	97.13	[6.3.9]
CoV2-05	IGHV3-20*04 F	IGHJ2*01 F	IGHD4-17*01 F	94.10	[8.8.14]	IGLV2-11*01 F	IGLJ3*02 F	96.53	[9.3.10]
★ CoV2-06	IGHV4-4*02 F	IGHJ4*02 F	IGHD6-13*01 F	94.44	[9.7.14]	IGLV2-14*01 F	IGLJ2*01 F	99.31	[9.3.10]
★ CoV2-09	IGHV4-34*01 F	IGHJ5*02 F	IGHD6-13*01 F	92.28	[8.7.18]	IGLV3-19*01 F	IGLJ2*01 F	92.47	[6.3.11]
CoV2-11	IGHV3-9*01 F	IGHJ4*02 F	IGHD3-22*01 F	95.83	[8.8.15]	IGLV3-21*04 F	IGLJ1*01 F	88.53	[6.3.11]
CoV2-12	IGHV3-30-3*01 F	IGHJ4*02 F	IGHD3-10*01 F	90.97	[8.8.16]	IGKV1-39*01 F	IGKJ2*01 F	99.28	[6.3.10]
CoV2-13	IGHV1-69*01 F	IGHJ4*02 F	IGHD3-10*01 F	90.28	[8.8.12]	IGKV1-17*01 F	IGKJ1*01 F	98.21	[6.3.9]
★ CoV2-14	IGHV6-1*01 F	IGHJ6*02 F	IGHD6-13*01 F	100.00	[10.9.18]	IGLV2-14*01 F	IGLJ2*01 F	96.53	[9.3.8]
★ CoV2-15	IGHV6-1*01 F	IGHJ6*02 F	IGHD6-13*01 F	100.00	[10.9.18]	IGLV2-8*01 F	IGLJ2*01 F	73.61	[9.3.9]
★ CoV2-16	IGHV4-34*01 F	IGHJ5*02 F	IGHD6-13*01 F	91.93	[8.7.18]	IGLV1-44*01 F	IGLJ1*01 F	89.47	[8.3.11]
★ CoV2-17	IGHV3-21*01 F	IGHJ3*02 F	IGHD5-12*01 F	99.31	[8.8.18]	IGLV1-47*01 F	IGLJ1*01 F	98.25	[8.3.11]
★ CoV2-18	IGHV1-69*06 F	IGHJ3*02 F	IGHD3-9*01 F	96.53	[8.8.19]	IGLV1-44*01 F	IGLJ1*01 F	94.74	[8.3.11]
CoV2-19	IGHV1-2*02 F	IGHJ4*02 F	IGHD3-16*01 F	96.53	[8.8.20]	IGKV1-39*01 F	IGKJ3*01 F	93.19	[6.3.9]
CoV2-20	IGHV3-9*01 F	IGHJ3*01 F	IGHD4-23*01 ORF	88.54	[8.8.14]	IGKV1-39*01 F	IGKJ5*01 F	94.62	[6.3.10]
CoV2-22	IGHV1-2*02 F	IGHJ4*02 F	IGHD3-16*01 F	97.22	[8.8.20]	IGKV1-39*01 F	IGKJ3*01 F	98.57	[6.3.9]
CoV2-23	IGHV3-9*01 F	IGHJ4*02 F	IGHD3-10*01 F	97.92	[8.8.14]	IGKV1-12*01 F	IGKJ2*01 F	94.27	[6.3.9]
CoV2-24	IGHV4-34*01 F	IGHJ3*02 F	IGHD1-26*01 F	91.58	[8.7.12]	IGLV3-21*02 F	IGLJ1*01 F	93.55	[6.3.11]
★ CoV2-25	IGHV1-2*06 F	IGHJ4*02 F	No result	99.31	[8.8.7]	IGLV1-44*01 F	IGLJ2*01 F	98.60	[8.3.11]
★ CoV2-26	IGHV1-2*06 F	IGHJ4*02 F	No result	99.31	[8.8.7]	IGLV1-44*01 F	IGLJ2*01 F	97.19	[8.3.11]
CoV2-27	IGHV4-34*01 F	IGHJ5*02 F	IGHD6-13*01 F	92.28	[8.7.18]	IGLV1-44*01 F	IGLJ3*02 F	94.74	[8.3.11]
CoV2-28	IGHV1-69*12 F	IGHJ3*02 F	IGHD1-26*01 F	99.65	[8.8.15]	IGLV2-14*01 F	IGLJ1*01 F	98.26	[9.3.10]
★ CoV2-29	IGHV3-7*03 F	IGHJ6*03 F	IGHD3-3*01 F	99.31	[8.8.19]	IGLV2-14*01 F	IGLJ2*01 F	98.96	[9.3.10]
CoV2-31	IGHV4-34*01 F	IGHJ5*02 F	IGHD2-2*01 F	93.68	[8.7.18]	IGLV3-9*01 F	IGLJ1*01 F	96.77	[6.3.9]
CoV2-32	IGHV3-9*01 F	IGHJ3*02 F	IGHD4-17*01 F	94.10	[8.8.16]	IGLV1-40*01 F	IGLJ1*01 F	98.61	[9.3.11]
CoV2-33	IGHV3-9*01 F	IGHJ6*02 F	IGHD3-10*01 F	96.53	[8.8.16]	IGLV2-14*01 F	IGLJ2*01 F	97.57	[9.3.11]
CoV2-34	IGHV4-34*01 F	IGHJ5*02 F	IGHD2-21*02 F	91.93	[8.7.18]	IGKV1-39*01 F	IGKJ5*01 F	91.76	[6.3.9]
CoV2-35	IGHV4-34*01 F	IGHJ5*02 F	IGHD6-13*01 F	93.68	[8.7.18]	IGLV3-21*04 F	IGLJ2*01 F	94.62	[6.3.10]

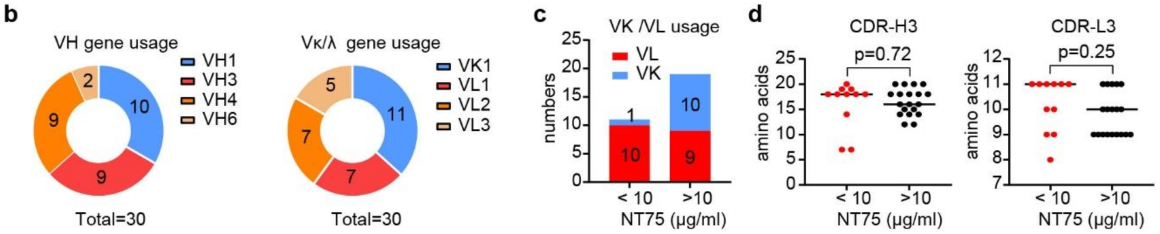

Supplementary Fig. 2. Germline gene origins, V-region identities, and the length of CDRs for the variable heavy and light chains. a Germline gene origins, V-region identities, and the length of CDRs for the variable heavy and light chains. The stars indicate the 11 antibodies with NT_{75}<10 μg/ml with the five most potent neutralizing antibodies marked with a red asterisk "*". **b** The germline gene classes for the variable heavy (VH) and the variable light chains (VK/L) of the SARS-CoV-2 antibodies. The numbers for each V gene used are indicated in the pie chart. **c** Comparison of the numbers of kappa light chain (VK) and lambda light chain (VL) used in the two groups of antibodies with NT_{75}<10 μg/ml and NT_{75}>10 μg/ml. **d** Comparison of the amino acid numbers of the heavy chain CDR3 (CDR-H3) and the light chain CDR3 (CDR-L3) in the two groups of SARS-CoV-2 antibodies with NT_{75}<10 μg/ml and NT_{75}>10 μg/ml. Two-tailed Student's t-test.

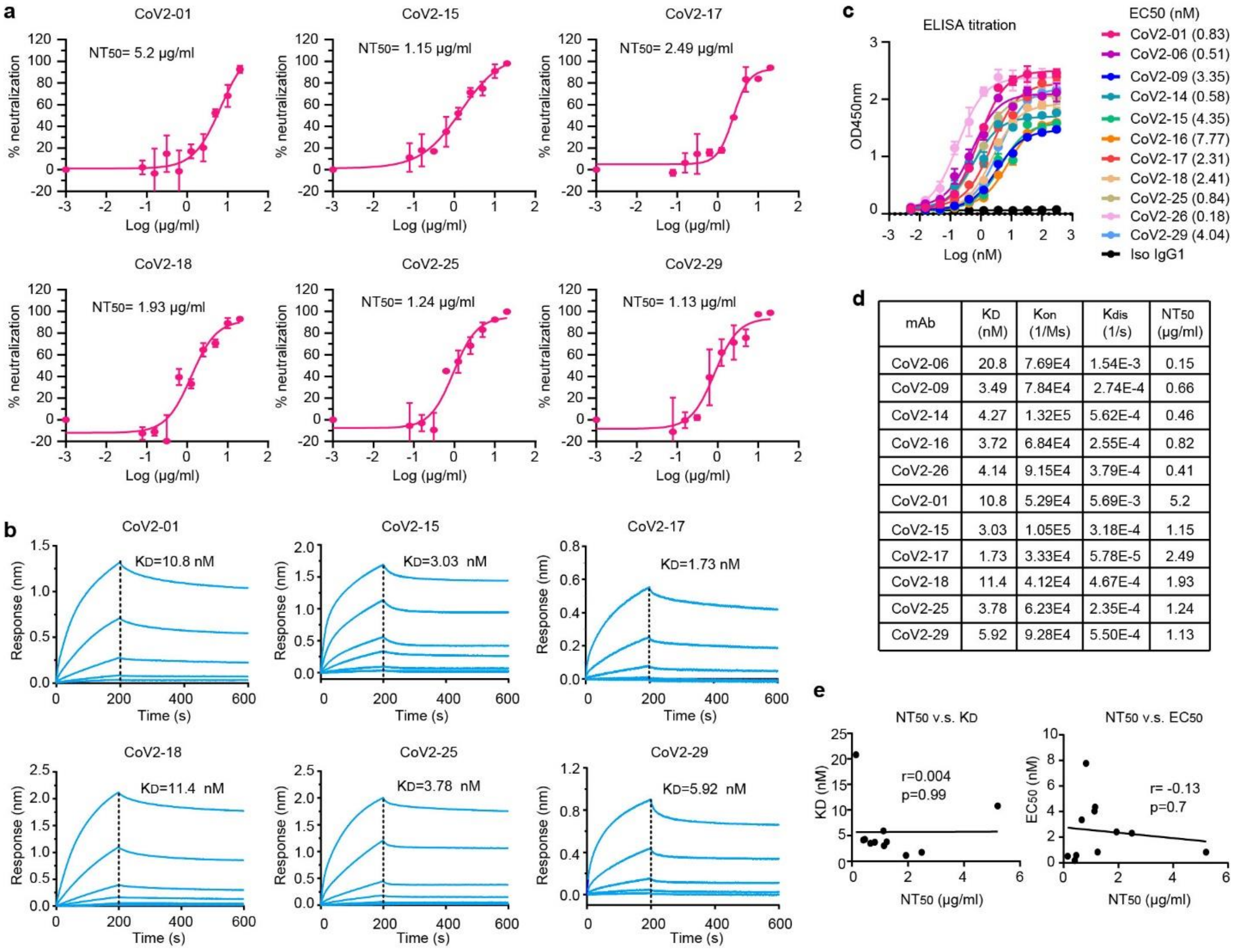

mAb	KD (nM)	Kon (1/Ms)	Kdis (1/s)	NT50 (μg/ml)
CoV2-06	20.8	7.69E4	1.54E-3	0.15
CoV2-09	3.49	7.84E4	2.74E-4	0.66
CoV2-14	4.27	1.32E5	5.62E-4	0.46
CoV2-16	3.72	6.84E4	2.55E-4	0.82
CoV2-26	4.14	9.15E4	3.79E-4	0.41
CoV2-01	10.8	5.29E4	5.69E-3	5.2
CoV2-15	3.03	1.05E5	3.18E-4	1.15
CoV2-17	1.73	3.33E4	5.78E-5	2.49
CoV2-18	11.4	4.12E4	4.67E-4	1.93
CoV2-25	3.78	6.23E4	2.35E-4	1.24
CoV2-29	5.92	9.28E4	5.50E-4	1.13

Supplementary Fig. 3. Neutralization, RBD binding and RBD/ACE2 blocking activities of additional mAbs. a Neutralization titration of the six remaining mAbs of the 11 mAbs with neutralizations above 75% at 10 μg/ml. Error bars indicate SD of duplicates. **b** Kinetic binding curves of the six mAbs to the RBD protein of SARS-CoV-2. **c** ELISA titration and determination of the 50% effective binding concentration (EC_{50}) of the 11 neutralizing antibodies to sCoV2-RBD. Each data point is the mean ± SD of two replicates. **d** A summary of the binding affinities (K_D), association constant (K_{on}), dissociation constant (K_{dis}), the 50% inhibition concentration (IC_{50}) of receptor blocking, and the NT_{50} of the 11 neutralizing antibodies. **e** The correlation between NT_{50} and the affinity K_D and between NT_{50} and the ELISA binding EC_{50} for each of the 11 mAbs. Pearson correlations were performed using Graphpad prism 8.

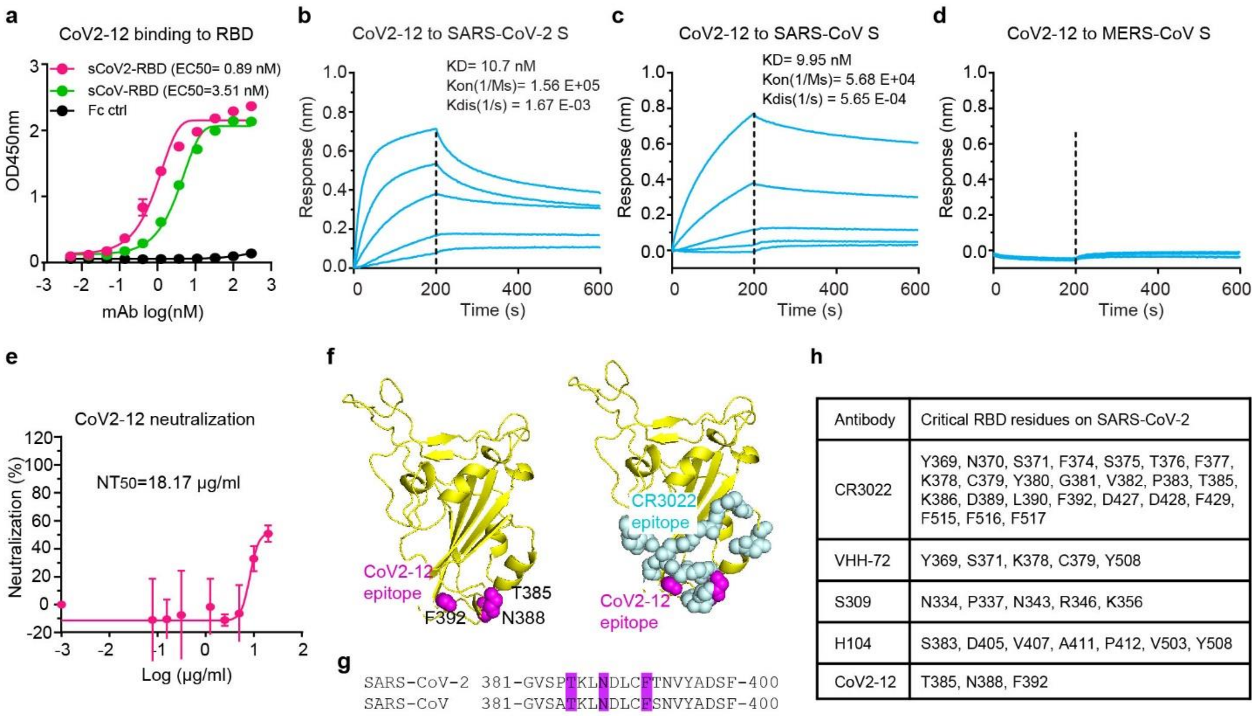

Antibody	Critical RBD residues on SARS-CoV-2
CR3022	Y369, N370, S371, F374, S375, T376, F377, K378, C379, Y380, G381, V382, P383, T385, K386, D389, L390, F392, D427, D428, F429, F515, F516, F517
VHH-72	Y369, S371, K378, C379, Y508
S309	N334, P337, N343, R346, K356
H104	S383, D405, V407, A411, P412, V503, Y508
CoV2-12	T385, N388, F392

Supplementary Fig. 4. A conserved epitope determined by the SARS-CoV-2 neutralizing and SARS-CoV cross-reactive mAb CoV2-12. a ELISA titration and determination of the 50% effective binding concentration (EC_{50}) of CoV2-12 to indicated the RBD proteins SARS-CoV and SARS-CoV-2. Data points are mean ± SD of two replicates. **b**-**d** Kinetic binding curves of CoV2-12 to the S protein of SARS-CoV-2 (**b**), SARS-CoV (**c**) and MERS-CoV (**d**). The dashed lines indicate the separation of association and dissociation phases. **e** SARS-CoV-2 neutralization titration of CoV-12. Data points are mean ± SD of two replicates. **f** The critical residues for CoV2-12 binding are shown as magenta spheres in the RBD structure and their locations relative to the epitope residues (colored in pale cyan) of the CR3022 mAb. **g** Alignment of the CoV2-12 binding residues on the RBD of SARS-CoV-2 and SARS-CoV. **h** Summary of the critical RBD residues on SARS-CoV-2 for CoV2-12 and the indicated cross-reactive mAbs.

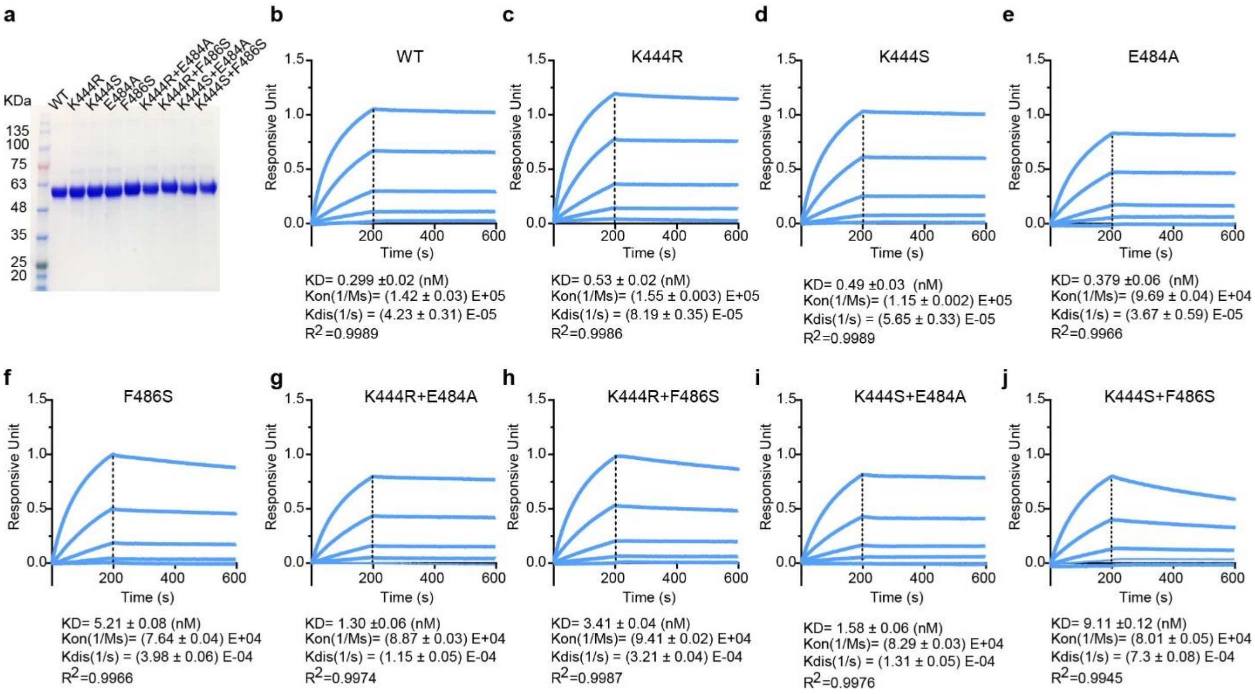

Supplementary Fig. 5. Expression of sCoV2-RBD mutant proteins and characterization of their affinities to ACE2. a SDS-PAGE and coomassie blue staining of the purified sCoV2-RBD proteins with indicated amino acid mutations. **b-j** Kinetic binding curves of the sCoV2-RBD wild type or mutant proteins to human ACE2.

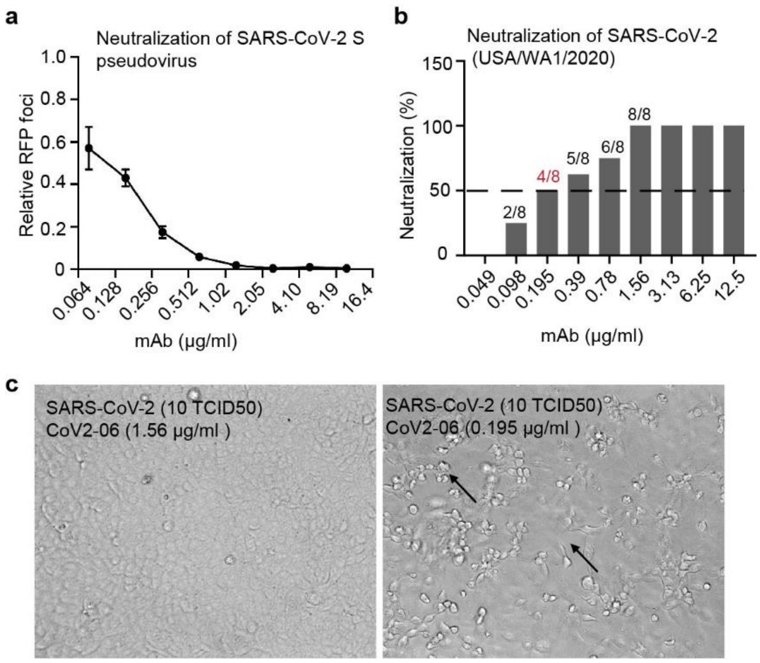

Supplementary Fig. 6. Validation of CoV2-06 neutralization against SARS-CoV-2 S pseudovirus and SARS-CoV-2 clinical isolate. a SARS-CoV-2 S pseudovirus neutralization assay with CoV2-06. The numbers of the red-fluorescent protein (RFP) focus were counted and their ratio relative to the control group without antibody were calculated and plotted as the Y-axis. Data points are mean ± SD of triplicates. **b** Percent neutralization of SARS-CoV-2 clinical isolate (USA/WA1/2020) infection as determined by visualizing the CPE of cells. The number of wells without CPE relative to the total 8 replicate wells shown on top of each bar. The dashed line indicates 50% of neutralization. **c** Representative images of protected and infected cells. The CPE, including cell detachment and syncytium, are indicated by arrows.

Mutation	CoV2-06	CoV2-14	CoV2-12	CoV2-09	CoV2-16	CoV2-26
T345A	21 (1)	123 (4)	40 (9)	56 (1)	33 (4)	103 (9)
R346A	1 (0)	93 (1)	81 (7)	145 (4)	158 (8)	106 (4)
S349A	48 (3)	60 (7)	42 (0)	6 (0)	2 (0)	52 (6)
T385A	91 (4)	101 (3)	11 (5)	115 (8)	113 (4)	97 (5)
N388A	68 (1)	74 (7)	23 (8)	74 (3)	58 (6)	72 (6)
F392A	86 (0)	103 (5)	7 (3)	112 (2)	114 (1)	93 (1)
K444A	2 (1)	102 (6)	135 (5)	91 (1)	96 (5)	105 (4)
G446A	7 (0)	82 (3)	74 (0)	2 (1)	1 (0)	56 (2)
G447A	1 (0)	97 (1)	66 (3)	0 (0)	1 (0)	85 (13)
N448A	21 (0)	49 (2)	55 (6)	0 (0)	0 (0)	31 (3)
Y449A	0 (0)	97 (3)	158 (7)	0 (0)	1 (1)	72 (3)
N450A	19 (1)	85 (2)	220 (36)	1 (0)	1 (0)	71 (7)
L452A	112 (0)	115 (1)	134 (3)	0 (0)	0 (0)	103 (2)
F456A	85 (1)	21 (2)	135 (4)	74 (0)	66 (2)	45 (2)
A475S	77 (4)	8 (1)	106 (0)	93 (7)	85 (0)	94 (9)
E484A	56 (1)	2 (0)	93 (4)	5 (0)	1 (0)	105 (2)
G485A	74 (4)	62 (1)	70 (3)	106 (0)	77 (3)	15 (9)
F486A	108 (2)	1 (0)	168 (2)	113 (2)	106 (2)	12 (4)
N487A	66 (0)	61 (1)	55 (2)	93 (1)	74 (0)	25 (2)
Y489A	74 (1)	1 (0)	91 (19)	52 (6)	52 (3)	10 (6)
F490A	86 (2)	75 (1)	123 (12)	0 (0)	1 (0)	109 (0)
G496A	88 (12)	102 (9)	143 (10)	12 (1)	4 (0)	104 (1)

Supplementary Table 1. Residues critical for mAb binding to SARS-CoV-2 S protein RBD. The mAb reactivity for each alanine scan mutant are expressed as percent of binding to wildtype S protein, with ranges (half of the maximum minus minimum values) in parentheses. At least two replicate values were obtained for each experiment. Values are shaded in grey for critical residues.

**Primers for constructing RBD expressing vectors**	**Forward primer**	**Reverse primer**
SCoV2-RBD	ACAGGTGTCCACTCGCTAGCTAGAGTGCAGCCTACCGAGA	GTGAACCGCCTCCACCTGCGCAGAAGTTCACGCATTTGTT
K444R overlap	AGCAACAACCTGGACAGCCGGGTCGGCGGCAAC	GTTGCCGCCGACCCGGCTGTCCAGGTTGTTGCT
K444S overlap	AGCAACAACCTGGACAGCTCCGTCGGCGGCAAC	GTTGCCGCCGACGGAGCTGTCCAGGTTGTTGCT
E484A overlap	ACCCCTTGCAATGGCGTGGCCGGCTTCAACTGCTACTTC	GAAGTAGCAGTTGAAGCCGGCCACGCCATTGCAAGGGGT
F486S overlap	TGCAATGGCGTGGAAGGCTCCAACTGCTACTTCCCACTG	CAGTGGGAAGTAGCAGTTGGAGCCTTCCACGCCATTGCA
N501A overlap	TACGGCTTCCAGCCTACAGCCGGCGTGGGCTACCAGCCT	AGGCTGGTAGCCCACGCCGGCTGTAGGCTGGAAGCCGTA
**Primers for scFv sequencing**	**Forward primer**	
PSCFVF	GAAATACCTGCTGCCGACTG	
**Primers for virus sequencing**	**Forward primer**	**Reverse primer**
cov-21521V	tgttatttctagtgatgttcttg	
cov-22092V	tggaccttgaaggaaaac	
cov-22685V	tccacttttaagtgttatggag	
cov-23203V	aggcacaggtgttcttac	
cov-23840V	gtacacaattaaaccgtgc	
cov-24428V	cacaagctttaaacacgc	
cov-25068V	tctctggcattaatgcttc	
cov-25238R		CAATCAAGCCAGCTATAAAACC
**Primers for virus construction**	**Forward primer**	**Reverse primer**
K444R SARS-CoV-2	ctaacaatcttgattctaGggttggtggtaattataattac	caccaaccCtagaatcaagattgttag
E484A SARS-CoV-2	gttgCaggttttaattgttactttcctttac	ggaaagtaacaattaaaacctGcaacaccattacaaggtg

Supplementary Table 2. Primers for gene cloning, virus sequencing, virus construction.
