# Supplementary material for: Molecular determinants and mechanism for antibody cocktail preventing SARS-CoV-2 escape
Source: Nat Commun. 2021 Jan 20;12:469. doi: 10.1038/s41467-020-20789-7 (PMC7817669; doi:10.1038/s41467-020-20789-7)
Supplement: Supplementary file 2 — Reporting Summary [file 41467_2020_20789_MOESM2_ESM.pdf]

nature research

Corresponding author(s): Zhiqiang An

Last updated by author(s): Dec 3, 2020

Reporting Summary

Nature Research wishes to improve the reproducibility of the work that we publish. This form provides structure for consistency and transparency in reporting. For further information on Nature Research policies, see our Editorial Policies and the Editorial Policy Checklist.

Statistics

For all statistical analyses, confirm that the following items are present in the figure legend, table legend, main text, or Methods section.

n/a	Confirmed	
☐	☒	The exact sample size (*n*) for each experimental group/condition, given as a discrete number and unit of measurement
☒	☐	A statement on whether measurements were taken from distinct samples or whether the same sample was measured repeatedly
☐	☒	The statistical test(s) used AND whether they are one- or two-sided<br>*Only common tests should be described solely by name; describe more complex techniques in the Methods section.*
☐	☒	A description of all covariates tested
☒	☐	A description of any assumptions or corrections, such as tests of normality and adjustment for multiple comparisons
☐	☒	A full description of the statistical parameters including central tendency (e.g. means) or other basic estimates (e.g. regression coefficient) AND variation (e.g. standard deviation) or associated estimates of uncertainty (e.g. confidence intervals)
☐	☒	For null hypothesis testing, the test statistic (e.g. *F*, *t*, *r*) with confidence intervals, effect sizes, degrees of freedom and *P* value noted<br>*Give P values as exact values whenever suitable.*
☒	☐	For Bayesian analysis, information on the choice of priors and Markov chain Monte Carlo settings
☒	☐	For hierarchical and complex designs, identification of the appropriate level for tests and full reporting of outcomes
☐	☒	Estimates of effect sizes (e.g. Cohen's *d*, Pearson's *r*), indicating how they were calculated

Our web collection on statistics for biologists contains articles on many of the points above.

Software and code

Policy information about availability of computer code

Data collection	Octet Data Acquisition 9.0 was used to collect affinity data, epitope binning data,RBD competition data and RBD expression data; SoftMax Pro 6.5.1 was used to collect ELISA data, UNICORN V7.0 was used to collect the SEC data; Gen5™ Microplate Reader and Imager was used to collect virus infection and neutralization data; Forecyt software (Standard Edition) was used to collect the flow cytometry data in epitope mapping.
Data analysis	Graphpad Prism 8; PyMOL V2.0.6; Octet Data Analysis software V11.1; UNICORN V7.0; CompuSyn (Version published in 2005); NCBI-BLAST V2.2.30; Orf Predictor (Version published in 2016); Clustal Omega V1.2.4

For manuscripts utilizing custom algorithms or software that are central to the research but not yet described in published literature, software must be made available to editors and reviewers. We strongly encourage code deposition in a community repository (e.g. GitHub). See the Nature Research guidelines for submitting code & software for further information.

Data

Policy information about availability of data

All manuscripts must include a data availability statement. This statement should provide the following information, where applicable:

- Accession codes, unique identifiers, or web links for publicly available datasets
- A list of figures that have associated raw data
- A description of any restrictions on data availability

All data generated and supporting the findings of this study are available within the paper or in the source data file.

Field-specific reporting

Please select the one below that is the best fit for your research. If you are not sure, read the appropriate sections before making your selection.

- [x] Life sciences
- [ ] Behavioural & social sciences
- [ ] Ecological, evolutionary & environmental sciences

For a reference copy of the document with all sections, see nature.com/documents/nr-reporting-summary-flat.pdf

Life sciences study design

All studies must disclose on these points even when the disclosure is negative.

Sample size	The group sizes for in vitro assays (e.g. ELISA, BLI, neutralization and flow cytometry) were selected based on prior knowledges in our previous publications (PMID: 33106671; 31213474). The group sizes for in vivo assays were selected on the basis of pilot studies and prior knowledges (PMID: 33106671).
Data exclusions	None
Replication	Replicates were used in the experiments as noted in the methods, figure legends and text.
Randomization	Mice were the same background , age- and gender-matched and randomized by animal research personnel at UTMB prior to experiment. Randomizations are irrlevant to in vitro cell line based assays or biochemical assays.
Blinding	No blinding was performed due to safety considerations regarding infected animals and cell culture.

Reporting for specific materials, systems and methods

We require information from authors about some types of materials, experimental systems and methods used in many studies. Here, indicate whether each material, system or method listed is relevant to your study. If you are not sure if a list item applies to your research, read the appropriate section before selecting a response.

Materials & experimental systems

n/a	Involved in the study	
[ ]	[x]	Antibodies
[ ]	[x]	Eukaryotic cell lines
[x]	[ ]	Palaeontology and archaeology
[ ]	[x]	Animals and other organisms
[x]	[ ]	Human research participants
[x]	[ ]	Clinical data
[x]	[ ]	Dual use research of concern

Methods

n/a	Involved in the study	
[x]	[ ]	ChIP-seq
[x]	[ ]	Flow cytometry
[x]	[ ]	MRI-based neuroimaging

Antibodies

Antibodies used	All the SARS-CoV-2 antibodies were generated in house<br>HRP-conjugated Mouse-anti-M13, Santa Cruz, #sc-53004 HRP, 1:1000<br>Goat Anti-Human IgG, F(ab')2 fragment specific, Jackson Immuno Research, #109-035-006, 1:5000<br>Mouse anti-VSV Indiana G, Clone 8G5F11, Absolute Antibody, 100ng/mL<br>Alexa Fluor 488-AffiniPure Goat Anti-Human IgG (H+L), Jackson ImmunoResearch, #109-545-003, 3.75 µg/mL
Validation	HRP-conjugated Mouse-anti-M13 was tested by the manufacture to demonstrate its ability to detect the phage M13 major coat protein ; The Mouse anti-VSV Indiana G antibody was tested by the manufacture and other studies to demonstrate its ability to detect cell-surface levels of VSV G-protein.

Eukaryotic cell lines

Policy information about cell lines

Cell line source(s)	Expi293 cells, Thermo Fisher, Cat#A14527; Vero-E6, ATCC, CRL-1586; HEK-293T, ATCC, CRL-3216
Authentication	The Expi 293, Vero-E6 and HEK-293T cells were previously reported but not authenticated by us.
Mycoplasma contamination	All cell lines were mycoplasma negative

Commonly misidentified lines (See ICLAC register)	none

Animals and other organisms

Policy information about studies involving animals; ARRIVE guidelines recommended for reporting animal research

Laboratory animals	Ten- to twelve-week old female BALB/c mice were purchased from Charles River Laboratories and maintained in SealsafeTM HEPA-filtered air in/out units
Wild animals	We did not used any wild animals
Field-collected samples	We did not collect any filed samples
Ethics oversight	Mice were maintained at University of Texas Medical Branch at Galveston in accordance with the guidelines of the Institutional Animal Care and use Committee (IACUC) under the protocol 1802011

Note that full information on the approval of the study protocol must also be provided in the manuscript.
